# Residual feed intake divergence during the preweaning period is associated with unique hindgut microbiome and metabolome profiles in neonatal Holstein heifer calves

**DOI:** 10.1186/s40104-019-0406-x

**Published:** 2020-01-20

**Authors:** Ahmed Elolimy, Abdulrahman Alharthi, Mohamed Zeineldin, Claudia Parys, Juan J. Loor

**Affiliations:** 1 Mammalian NutriPhysioGenomics, Department of Animal Sciences, University of Illinois, Urbana, IL USA; 2 Department of Animal Sciences, University of Illinois, Urbana, IL USA; 3 Department of Animal Production, National Research Centre, Dokki, Giza, Egypt; 4 Carl R. Woese Institute for Genomic Biology, University of Illinois, Urbana, Illinois USA; 5 Department of Animal Medicine, College of Veterinary Medicine, Benha University, Benha, Egypt; 6 Evonik Nutrition & Care GmbH, Hanau-Wolfgang, Germany; 7 Division of Nutritional Sciences, Illinois Informatics Institute, University of Illinois, Urbana, IL USA

**Keywords:** Feed efficiency, Gut, Metabolomics, Microbiota, Neonates, Newborn, Preweaning, RFI

## Abstract

**Background:**

Recent studies underscored that divergence in residual feed intake (RFI) in mature beef and dairy cattle is associated with changes in ruminal microbiome and metabolome profiles which may contribute, at least in part, to better feed efficiency. Because the rumen in neonatal calves during the preweaning period is underdeveloped until close to weaning, they rely on hindgut microbial fermentation to breakdown undigested diet components. This leads to production of key metabolites such as volatile fatty acids (VFA), amino acids, and vitamins that could potentially be absorbed in the hind-gut and help drive growth and development. Whether RFI divergence in neonatal calves is associated with changes in hindgut microbial communities and metabolites is largely unknown. Therefore, the objective of the current study was to determine differences in hindgut microbiome and metabolome in neonatal Holstein heifer calves retrospectively-grouped based on feed efficiency as most-efficient (M-eff) or least-efficient (L-eff) calves using RFI divergence during the preweaning period.

**Methods:**

Twenty-six Holstein heifer calves received 3.8 L of first-milking colostrum from their respective dams within 6 h after birth. Calves were housed in individual outdoor hutches bedded with straw, fed twice daily with a milk replacer, and had ad libitum access to a starter grain mix from birth to weaning at 42 d of age. Calves were classified into M-eff [*n* = 13; RFI coefficient = − 5.72 ± 0.94 kg DMI (milk replacer + starter grain)/d] and L-eff [*n* = 13; RFI coefficient = 5.61 ± 0.94 kg DMI (milk replacer + starter grain)/d] based on a linear regression model including the combined starter grain mix and milk replacer DMI, average daily gain (ADG), and metabolic body weight (MBW). A deep sterile rectal swab exposed only to the rectum was collected immediately at birth before colostrum feeding (i.e., d 0), and fecal samples at d 14, 28, and 42 (prior to weaning) for microbiome and untargeted metabolome analyses using 16S rRNA gene sequencing and LC-MS. Microbiome data were analyzed with the QIIME 2 platform and metabolome data with the MetaboAnalyst 4.0 pipeline.

**Results:**

No differences (*P* > 0.05) in body measurements including body weight (BW), body length (BL), hip height (HH), hip width (HW), and wither height (WH) were detected between M-eff and L-eff calves at birth and during preweaning. Although milk replacer intake did not differ between groups, compared with L-eff, M-eff heifers had lower starter intake (*P* < 0.01) between d 18 to 42 of age, whereas no differences (*P* > 0.05) for ADG, cumulative BWG, or body measurements were observed between RFI groups during the preweaning period. Microbiome and metabolome profiles through the first 42 d of age indicated greater hindgut capacity for the production of energy-generating substrates (butyrate and propionate) and essential nutrients (vitamins and amino acids) in heifers with greater estimated feed efficiency.

**Conclusion:**

Despite consuming approximately 54.6% less solid feed (cumulative intake, 10.90 vs. 19.98 ± 1.66 kg) from birth to weaning, the microbiome-metabolome changes in the hindgut of most-efficient heifers might have helped them maintain the same level of growth as the least-efficient heifers.

## Background

In dairy farming systems, feed costs account for approximately 60% of production expenses [1]. Therefore, identifying biological regulators of feed-efficiency in young dairy cattle would maximize profit margins [2]. The RFI is a relatively new measurement of feed efficiency in dairy cattle [3, 4], and is defined as the difference between actual and predicted feed intake, whereby predicted intake is calculated using a linear regression of actual intake on metabolic body weight (BW^0.75^) and average daily gain (ADG) [5]. The most-efficient animals (M-eff) have actual intakes smaller than predicted resulting in negative RFI coefficients, whereas the opposite is true for least-efficient animals (L-eff). In a previous study involving 2000 dairy heifer calves, compared with L-eff heifer calves, Macdonald et al. [6] observed that M-eff heifers selected according to RFI ranking at 6 months of age maintained superior feed efficiency (i.e. negative RFI coefficient) during the first lactation at 29 months of age. Clearly, approaches to identify and select for M-eff heifers in early life could be a useful tool for reducing feeding costs and maximizing profit margins.

Although the biological mechanisms driving RFI divergence are not fully understood, alterations in ruminal microbiome and metabolome profiles in adult cattle are associated with RFI ranking. For instance, M-eff cows had greater total bacterial density including fibrolytics (*Fibrobacter succinogenes*) around parturition [7], and abundance of bacterial genera *Anaerovibrio* and *Butyrivibrio* also was greater in established lactation [8]. Those data suggested that, compared with L-eff cattle, changes in ruminal bacteria in M-eff cattle might contribute, at least in part, to better rates of digestibility of dry matter, organic matter, and neutral detergent fiber [9]. Other studies detected greater concentrations of energy-related metabolites in the rumen of M-eff lambs and dairy cows including butyrate and propionate [10, 11], suggesting a contribution of these microbial-derived compounds to energy metabolism and milk production [12]. Unlike mature ruminants, neonatal calves have an undeveloped rumen until close to weaning. Therefore, undigested diet components reach the hindgut where microbial metabolism produces numerous compounds such as volatile fatty acids (VFA), amino acids, and vitamins that help regulate neonatal growth and development [13]. Whether differences in hindgut microbiome and metabolome contribute to RFI divergence in dairy calves, as in mature cows, during the preweaning period remains largely unknown.

The general hypothesis was that divergence in RFI during the preweaning period is associated with differences in hindgut microbiome and metabolome. The main objective of this study was to use deep sterile rectal swabs at birth and fecal samples through weaning along with individual measures of growth and development to evaluate the potential role of the hindgut in determining feed efficiency in young calves [14].

## Methods

The research protocol was approved by the Institutional Animal Care and Use Committee of the University of Illinois (Protocol No. 14270).

### Enrolment criteria and management of neonatal heifers

Immediately after parturition, newborn Holstein heifer calves were separated from their dams. Calves were kept in the experiment if they fulfilled all the following criteria described previously by Jacometo et al. [15]: (1) single heifer calf; (2) heifer calf birth weight > 36 kg; (3) calving difficulty score < 3; (4) dam first colostrum volume > 3.8 L; and (5) dam first colostrum IgG content > 50 mg/L. A subset of calves (*n* = 26; BW at birth = 42.0 ± 4.8 kg, mean ± SD) were selected randomly for the current study. All heifer calves were managed in the same fashion during the first 6 weeks of life. At birth, the navel was disinfected with 7% tincture of iodine solution (First Priority Inc., Elgin, IL, USA), and calves were vaccinated with TSV II (Pfizer Inc., New York, NY, USA) via nostril application. Calves received 3.8 L of first-milking colostrum collected from their dams within 6 h after birth. Heifers were housed in individual outdoor hutches bedded with straw, and fed twice daily (morning and afternoon) with a milk replacer (Advance Excelerate, Milk Specialties, Carpentersville, IL, USA; 28.5% CP, 15% fat) until 35 d of age. The nutrient composition and amino acid profiles of the milk replacer are reported in Additional file 2: Table S1.

At d 36, neonatal heifers were switched to once-daily milk replacer feeding in the morning until weaning (42 d of age). Calves received 4.54 kg/d of milk replacer mix (0.59 kg of milk replacer in 3.95 L of water) from 1 to 10 d of age, 5.90 kg/d (0.77 kg of milk replacer in 5.13 L of water) from 11 to 20 d of age, 7.26 kg/d (0.94 kg of milk replacer in 6.32 L of water) from 21 to 35 d of age and 3.63 kg/d (0.47 kg of milk replacer in 3.16 L of water) from 36 to 42 d of age. All heifer calves consumed the milk replacer offered daily. From d 1 until 42 of life, neonatal heifers had ad libitum access to a starter grain mix (Ampli-Calf Starter 20®; 19.9% crude protein (CP) and 13.5% neutral detergent fiber (NDF), Purina Animal Nutrition, Shoreview, MN, USA) fed in the morning. The nutrient composition and amino acid profiles of the starter grain mix are reported in Additional file 2: Table S1. Starter grain mix intake and milk replacer intake were recorded daily for each calf until 42 d of age. Body measurements including BW (Toledo Floor Digital Scale model 2191, Mettler Toledo, Columbus, OH, USA), HH and HW, WH and BL were measured at d 0 (i.e., at birth before colostrum feeding), 7, 14, 21, 28, 35 and 42 before feeding the starter grain mix in the morning. Average daily gain (ADG) was calculated as final body weight (BW) at d 42 minus initial BW at birth divided by total number of days on trial (i.e. 42). Cumulative body weight gain (BWG) was calculated as BW at d 42 minus initial BW at birth. Average daily gain per week was calculated as final BW at the end of the week minus initial BW at the beginning of the week divided by total number of days per week (i.e. 7).

### Rectal and fecal sampling and storage

Rectal samples at birth were obtained from each heifer before colostrum feeding using sterile double sheathed equine uterine culture swabs (EquiVet, Kruuse, Denmark) inserted 10 cm into the rectum. The swab was only exposed to the rectum. For fecal samples at d 14, 28 and 42, calves were rectally finger-stimulated with a sterile-gloved hand to facilitate the collection of fresh feces that was subsequently placed in a sterile Whirl-Pak® bag (Nasco, Fort Atkinson, WI, USA). Rectal swabs and fecal bags were immediately flash frozen in liquid nitrogen and stored at − 80 °C for microbiome and metabolome analyses.

### Residual feed intake calculation

The RFI was calculated using the PROC MIXED procedure of SAS procedure of SAS 9.4 (SAS Institute Inc., Cary, NC, USA). An RFI coefficient was calculated for each individual heifer calf throughout the entire preweaning period from birth to 42 d of age, and assumed to represent the residuals from a multiple regression model regressing the combined DMI of starter grain mix and milk replacer on ADG and mid-test metabolic BW (MMW, i.e. BW^0.75^): predicted DMI = *β*_0_ + (*β*_1_ × ADG) + (*β*_2_ × MMW) + *ɛ*, in which *β*_0_ is the y-intercept, *β*_1_ is the partial regression coefficient of ADG, *β*_2_ is the partial regression coefficient of MMW, and *ɛ* is the error term. The RFI coefficient (kg DMI/d) for each individual heifer was then calculated as the difference between actual and predicted DMI [16]. The coefficient of determination (*R*^2^) was 0.76.

**Fig. 1:**
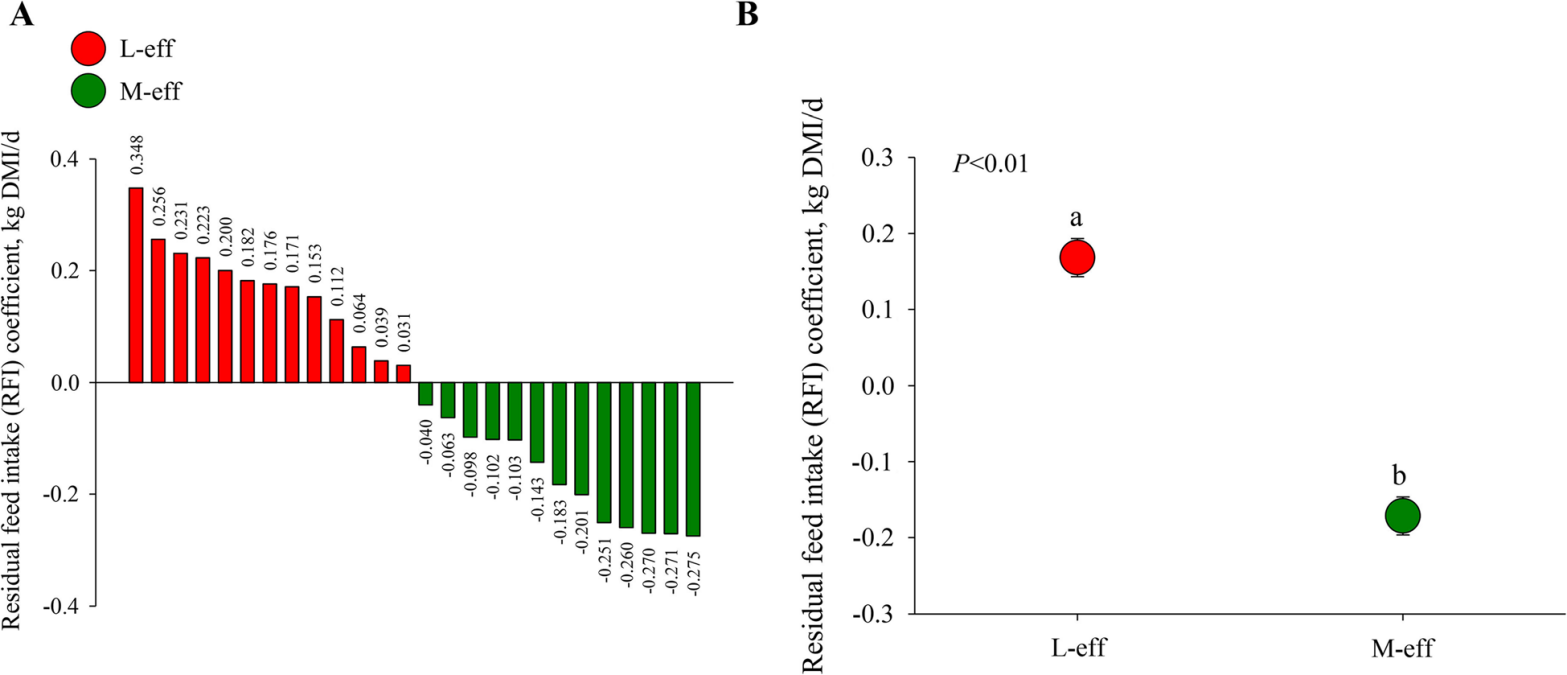
Residual feed intake (RFI) in least-efficient (L-eff, *n* = 13) or most-efficient (M-eff, *n* = 13) heifer calves during the preweaning period**. a** RFI population distribution. **b** RFI coefficients of the trial cohort (26 heifer calves)

All heifers were ranked by RFI, allowing the formation of two groups based on divergence in RFI: L-eff group with an unfavorable (i.e. more positive) RFI coefficient (*n* = 13) and M-eff group with a desirable (i.e. more negative) RFI (*n* = 13). The distribution and overall RFI coefficients for L-eff and M-eff groups are depicted in Fig. 1a and Fig. 1b.

### Rectal and fecal DNA extraction, 16S rRNA gene amplification and sequencing

Microbial DNA was extracted from rectal and fecal samples (single fecal swab or 100 mg feces from fecal bags) using DNeasy PowerSoil kit (Qiagen, Valencia, CA, USA) in accordance with manufacturer’s instructions. To track any contamination during the DNA extraction, 3 no-template negative controls (i.e. samples without biological material) were processed to assess the presence of microbial contamination in the swabs and the extraction reagents. The negative controls were run through the entire workflow alongside samples for quality control. Total DNA concentration and integrity were evaluated using NanoDrop spectrophotometer (ND 1000, NanoDrop Technologies, Inc., Wilmington, DE, USA) and 2% (wt/v) agarose gel electrophoresis (Sigma-Aldrich, Saint Louis, MO, USA) with SYBR Safe DNA Gel Stain (Invitrogen, Grand Island, NY, USA). The extracted DNA was immediately stored at − 80 °C for further analysis. All DNA samples were quantified on a Qubit fluorometer (Life technologies, Grand Island, NY, USA) using High Sensitivity DNA Kit (Roche, Indianapolis, IN, USA) and 20x Access Array loading reagent as described by Zeineldin et al. [17]. Total metagenomic DNA was subjected to Fluidigm Access Array Amplification (Fluidigm Corporation, South San Francisco, CA, USA) for DNA amplification. The V3-V4 hyper-variable region of 16S rRNA gene was sequenced with the Illumina MiSeq V2 platform (Illumina, San Diego, CA, USA) to obtain paired-end reads of 250 bp [18]. Data quality filters on the raw microbiome sequences were applied with Illumina software. Any reads found in the negative control were filtered out of the data analysis.

### Analysis of amplicon sequencing data

High quality 16S rRNA amplicon sequences were analyzed with open source Quantitative Insights into Microbial Ecology (QIIME) 2.0. The reads were de-noised into amplicon sequence variants (ASVs) using the DADA2 pipeline, an tool available in QIIME 2.0. Taxonomic classification of sequences was assigned to ASVs using the feature classifier against SILVA ribosomal RNA gene database. Singletons were removed prior to downstream analyses.

Bacterial alpha diversity, including Shannon, Chao1 and observed species indices per sample were calculated with QIIME 2.0. We visualized differences in beta-diversity with non-metric multidimensional scaling (NMDS) plots, which were constructed using MicrobiomeAnalyst [19]. Hindgut microbiome profiles were described for the most prevalent taxa using taxonomy plots generated in JMP 13.2 (SAS Institute Inc., Cary, NC, USA). Cladogram of LEfSe analysis for overrepresented microbes between L-eff and M-eff groups was obtained through Galaxy workflow framework. The PICRUSt 1.1.2 (Phylogenetic Investigation of Communities by Reconstruction of Unobserved States) pipeline and STAMP 2.1.3 were used to investigate and illustrate alterations in microbial functions of the hindgut microbiome associated with RFI divergence.

### Rectal and fecal metabolite extraction and LC-MS analysis

Free metabolites were extracted as described by Yu et al. [20] with modifications. Rectal and fecal samples (single fecal swab or 100 mg feces from fecal bags) were dissolved in 1 mL ice cold purified water prepared in a Milli-Q water purification system (Millipore Corp, Bedford, MA, USA). The mixture was vortexed and centrifuged at 10,000×*g* for 15 min at 4 °C. Supernatant was collected and kept on ice, whereas the remaining fecal pellet was further extracted by adding 1 mL ice cold LC-MS grade methanol (Sigma-Aldrich, Steinheim, Germany). The mixture was vortexed and centrifuged at 10,000×*g* for 15 min at 4 °C. Supernatant was collected and kept on ice. Both fecal supernatants were combined and centrifuged at 10,000×*g* for 15 min at 4 °C. The resulting supernatant was collected and stored at − 80 °C until LC-MS analysis.

Samples were analyzed with Q-Exactive MS system (Thermo. Bremen, Germany) in the Metabolomics Laboratory of Roy J. Carver Biotechnology Center, University of Illinois at Urbana-Champaign, USA. Software Xcalibur 4.1.31.9 was used for data acquisition. The Dionex Ultimate 3000 series HPLC system (Thermo, Germering, Germany) used included a degasser, an autosampler and a binary pump. The LC separation was performed on a Phenomenex Kinetex C18 column (4.6 mm × 100 mm, 2.6 μm) with mobile phase A (H_2_O with 0.1% formic acid) and mobile phase B (acetonitrile with 0.1% formic acid). The flow rate was 0.25 mL/min. The linear gradient was as follows: 0-3 min, 100% A; 20-30 min, 0% A; 31-36 min, 100% A. The autosampler was set to 15 °C. The injection volume was 20 μL. Mass spectra were acquired under both positive (sheath gas flow rate: 45; aux gas flow rate: 11; sweep gas flow rate: 2; spray voltage: 3.5 kV; capillary temp: 250 °C; Aux gas heater temp: 415 °C) and negative electrospray ionization (sheath gas flow rate: 45; aux gas flow rate: 11; sweep gas flow rate: 2; spray voltage: − 2.5 kV; capillary temp: 250 °C; Aux gas heater temp: 415 °C). The full scan mass spectrum resolution was set to 70,000 with scan range of *m/z* 67 ~ *m/z* 1000, and AGC target was 1E6 with a maximum injection time of 200 ms. 4-Chloro-DL-phenylalanine was spiked into samples as the internal standard. LC-MS data were further analyzed with Thermo Compound Discoverer software (v. 2.1 SP1) for chromatographic alignment and compound/feature identification/quantitation. The workflow used was Untargeted Metabolomics with Statistics Detect Unknowns with ID Using Online Databases. The following settings were used in Select Spectra: minimum precursor mass (65 Da) and maximum precursor mass (5000 Da); in Align Retention Time: Maximum shift (1 min) and Mass tolerance (5 ppm); in Detect unknown compounds: Mass tolerance (5 ppm), Intensity tolerance (30%), S/N (3), and Minimum peak intensity (1000000).

### Metabolomics data processing

Data visualization and statistical analyses of hindgut metabolome data were performed with MetaboAnalyst 4.0 [21]. The raw data were checked for data integrity and normalized by sum and autoscaling in order to enhance performance for downstream statistical analysis. Multivariate analysis was performed by the supervised partial least squares discriminant analysis (PLS-DA) to visualize metabolic profile dissimilarities between L-eff and M-eff groups in order to identify important metabolites separating the two groups and trends in upregulation or downregulation in the M-eff group. Metabolites most strongly influencing discrimination between M-eff and L-eff groups were selected according to their importance in differentiating the metabolic profiles based on the following criteria: variable importance in the projection (VIP) score > 1.0 and |p-(corr)| ≥ 0.5 with 95% jack-knifed confidence intervals. The confidence level 3 of Metabolomics Standards Initiative, i.e. annotate metabolites against a single parameter such as molecular weight (MW) [22], was used to annotate the differentially expressed metabolites according to accurate MW by searching the exact MW against the online Human Metabolome Database (HMDB) version 4.0 and Kyoto Encyclopedia of Genes and Genomes (KEGG) database. Differentially expressed metabolites identified from the above approach were used to perform pathway enrichment analysis using MetaboAnalyst 4.0 to explore upregulated and downregulated metabolic pathways in which the differential metabolites are involved in order to obtain an accurate insight into the underlying biology of the differentially expressed metabolites [21].

### Statistical analysis

The Shapiro-Wilks test in SAS 9.4 (SAS Institute Inc., Cary, NC, USA) was used to check normality of body measurements at birth and during the preweaning period. The UNIVARIATE procedure in SAS 9.4 was used for body measurements between L-eff and M-eff groups at birth, cumulative DMI and cumulative BWG. The MIXED procedure in SAS 9.4 was used for repeated measures analysis of body measurements, daily DMI and ADG at d 14, 28 and 42 of age. Both RFI groups and time (day or week) were considered as fixed factors in the model, and the random effect was calf. Comparison of bacterial alpha diversity indices in hindgut microbial communities between L-eff and M-eff groups at birth was performed with the nonparametric Mann-Whitney unpaired t-test with JMP 13.2 (SAS Institute Inc., Cary, NC, USA). Permutational multivariate analysis of variance (PERMANOVA) utilizing a Bray-Curtis dissimilarity index, a non-parametric multivariate analysis of variance was run in JMP 13.2 (SAS Institute Inc., Cary, NC, USA) to determine differences in bacterial alpha diversity indices between RFI groups at d 14, 28 and 42. Linear discriminant analysis (LDA) effect size (LEfSe) analysis was used to identify the differential genera between L-eff and M-eff groups. Significance was determined at *P* ≤ 0.05.

## Results

### Body measurements and growth performance

**Fig. 2:**
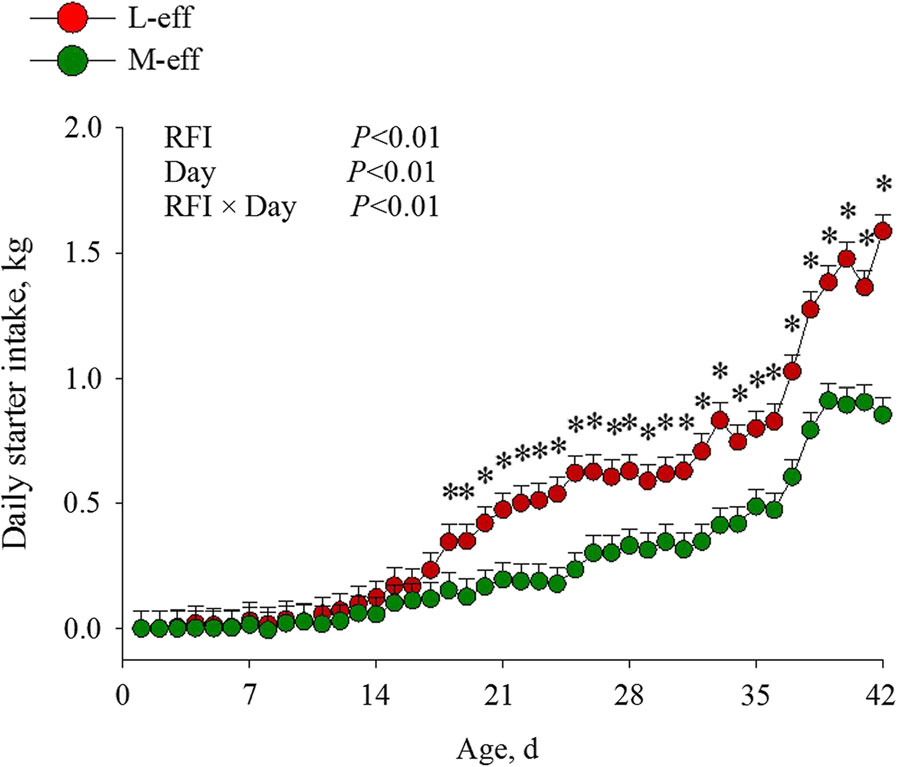
Daily starter dry matter intake (DMI) in least-efficient (L-eff, *n* = 13) or most-efficient (M-eff, *n* = 13) heifer calves

The Shapiro-Wilks test indicated that body measurements at birth and during the preweaning period for the 26 calves at each individual time point were normally distributed (*P* > 0.05). The difference in RFI coefficient between M-eff and L-eff was 0.32 kg DMI/d (Fig. 1a and Fig. 1b). No differences in body measurements at birth were detected (Table 1). During the preweaning period, M-eff heifers consumed less (*P* < 0.01) starter DMI between d 18 to d 42 (Fig. 2), whereas no differences (*P* > 0.05) for ADG, cumulative BWG, or body measurements were observed between RFI groups during the preweaning period (Table 2).

**Table 1:** Body measurements at birth in least-efficient (L-eff, *n* = 13) or most-efficient (M-eff, *n* = 13) heifer calves

Body measurement	L-eff	M-eff	SEM^1^	*P*-value
Body weight, kg	43.69	41.15	1.21	0.12
Body length, cm	109.5	109.7	1.66	0.92
Hip height, cm	80.08	80.58	1.00	0.71
Hip width, cm	16.28	15.63	0.42	0.29
Wither height, cm	76.41	77.24	0.97	0.52

^1^Standard error of the mean

^a,b^Different letters indicate significant differences due to the main maternal effect (*P* < 0.05)

**Table 2:** Body measurements and growth performance during preweaning period in least-efficient (L-eff, *n* = 13) or most-efficient (M-eff, *n* = 13) heifer calves

Week	RFI		SEM^1^	*P*-value		
	L-eff	M-eff		RFI	Time	RFI × Time
Weekly body weight, kg						
1	43.62	42.31	1.36	0.30	< 0.01	0.11
2	46.25	44.91	1.36			
3	50.38	49.48	1.36			
4	56.39	54.69	1.36			
5	61.76	59.72	1.36			
6	67.41	63.38	1.36			
Weekly body length, cm						
1	111.82	111.37	1.59	0.89	< 0.01	0.90
2	114.81	115.92	1.59			
3	117.99	118.31	1.59			
4	121.18	122.19	1.59			
5	125.17	125.01	1.59			
6	128.54	128.47	1.59			
Weekly hip height, cm						
1	81.42	81.90	0.82	0.98	< 0.01	0.40
2	83.06	83.52	0.82			
3	84.57	84.30	0.82			
4	85.88	85.36	0.82			
5	87.45	87.49	0.82			
6	89.54	89.18	0.82			
Weekly hip width, cm						
1	16.49	16.16	0.32	0.11	< 0.01	0.34
2	17.74	17.61	0.32			
3	18.88	18.18	0.32			
4	19.56	18.84	0.32			
5	20.36	19.60	0.32			
6	21.33	20.36	0.32			
Weekly wither height, cm						
1	77.50	77.80	0.80	0.81	< 0.01	0.53
2	78.29	79.25	0.80			
3	80.54	80.60	0.80			
4	81.64	81.64	0.80			
5	83.25	83.45	0.80			
6	85.08	85.14	0.80			
Weekly body weight gain^2^, kg/d						
1	0.10^b^	0.37^a^	0.10	0.94	< 0.01	< 0.01
2	0.38	0.37	0.10			
3	0.59	0.65	0.10			
4	0.86	0.75	0.10			
5	0.77	0.72	0.10			
6	0.91^a^	0.52^b^	0.10			
Cumulative body weight gain, kg	23.83	23.68	0.50	0.94		
Cumulative starter DMI, kg	19.98	10.90	1.66	< 0.01		

^1^Standard error of the mean

^2^Average daily gain per week (kg) = (final BW – initial BW)/7

^a,b^Different letters indicate significant differences due to the main maternal effect (*P* < 0.05)

### Hindgut microbiome at birth

**Fig. 3:**
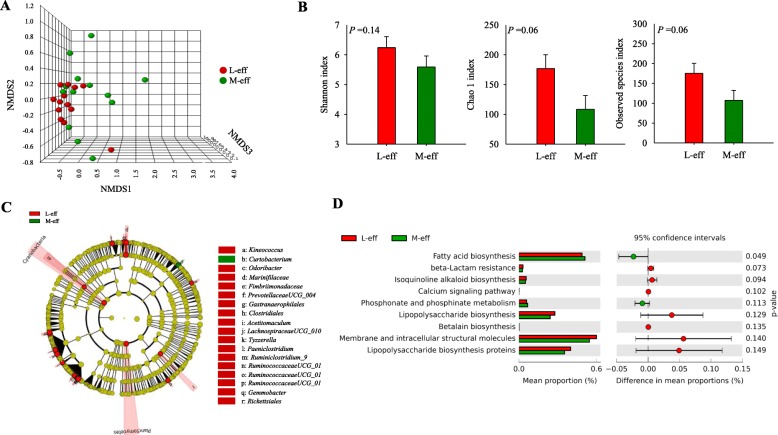
Hindgut microbiome at birth in least-efficient (L-eff, *n* = 13) or most-efficient (M-eff, *n* = 13) heifer calves. **a** Non-metric multidimensional scaling (NMDS) plot of fecal microbiome profiles. **b** Alpha diversity indices. **c** Cladogram of LEfSe analysis shows the overrepresented microbial populations. Taxa were significant when assessed by LeFSe (*P* ≤ 0.05 and LDA cutoff > 3.0). **d** Microbial functional predictions revealed the most differentially regulated metabolic pathways in the fecal microbiome at KEGG level 3 (i.e. Environmental Information Processing)

No statistical differences were detected between RFI groups at birth in beta diversity of microbial communities with the NMDS approach (ANalysis Of SIMilarity (ANOSIM); *P* = 0.20) (Fig. 3a and Additional file 1: Figure S1). This was further confirmed by the lack of difference in Shannon (*P* = 0.14), Chao 1 (*P* = 0.06) and observed species (*P* = 0.06) diversity indices (Fig. 3b). However, LeFSe analysis revealed shifts in hindgut microbiome communities at birth (Fig. 3c and Table 3). For example, M-eff heifers had greater abundance (*P* ≤ 0.05 and LDA cutoff > 2.0) of *Curtobacterium* but lower (*P* ≤ 0.05 and LDA cutoff > 2.0) *Kineococcus*, *Odoribacter*, Marinifilaceae, Fimbriimonadaceae, *Prevotellaceae UCG-004*, Gastranaerophilales, Clostridiales, *Acetitomaculum*, *Lachnospiraceae UCG-010*, Tyzzerella, *Paeniclostridium*, *Ruminiclostridium 9*, *Ruminococcaceae UCG-010*, *Ruminococcaceae UCG-014, Ruminococcaceae UCG-013, Gemmobacter*, and *Rickettsiales* (Fig. 3c and Table 3). In addition, the M-eff microbiome had greater number of functional genes (*P* ≤ 0.05) involved in VFA biosynthesis (Fig. 3d).

**Table 3:** Relative abundance (%) of the most differentially abundant bacteria highlighted by LeFSe analysis (*P* ≤ 0.05 and LDA cutoff > 2.0) in sterile rectal swabs at birth in least-efficient (L-eff, *n* = 13) or most-efficient (M-eff, *n* = 13) heifer calves

Bacteria	L-eff	M-eff
g_*Kineococcus*	1.117^a^	0.001^b^
g_*Curtobacterium*	0.250^b^	0.370^a^
g_*Odoribacter*	0.668^a^	0.130^b^
f_Marinifilaceae	0.542^a^	0.223^b^
f_Fimbriimonadaceae	0.567^a^	0.102^b^
g_*Prevotellaceae UCG-004*	0.233^a^	0.149^b^
o_Gastranaerophilales	0.595^a^	0.170^b^
o_Clostridiales	0.357^a^	0.057^b^
g_*Acetitomaculum*	2.220^a^	0.019^b^
g_*Lachnospiraceae UCG-010*	0.648^a^	0.175^b^
g_*Tyzzerella 4*	0.542^a^	0.454^b^
g_*Paeniclostridium*	0.340^a^	0.005^b^
g_*Ruminiclostridium 9*	0.251^a^	0.064^b^
g_*Ruminococcaceae UCG-010*	1.119^a^	0.110^b^
g_*Ruminococcaceae UCG-014*	0.184^a^	0.055^b^
g_*Ruminococcaceae UCG-013*	0.797^a^	0.239^b^
g_*Gemmobacter*	0.316^a^	0.000^b^
o_Rickettsiales	0.045^a^	0.000^b^

^a,b^Different letters indicate significant differences due to the main maternal effect (*P* < 0.05)

*o* order, *f* family, *g* genus

### Hindgut microbiome during the preweaning period

**Fig. 4:**
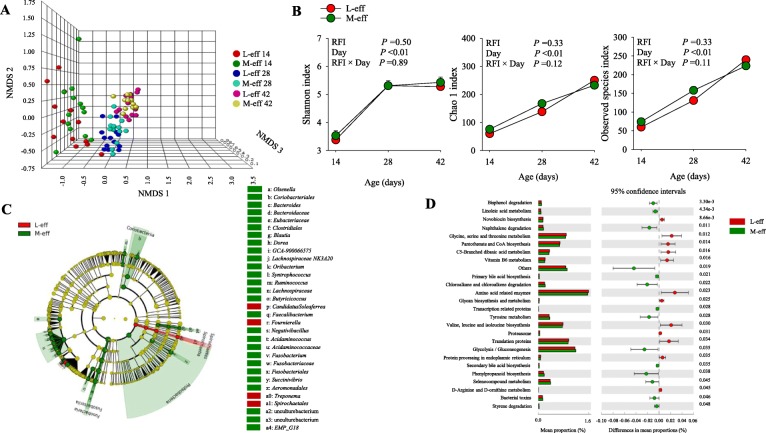
Hindgut microbiome during the preweaning period in least-efficient (L-eff, *n* = 13) or most-efficient (M-eff, *n* = 13) heifer calves. **a** Non-metric multidimensional scaling (NMDS) plot of fecal microbiome profiles. **b** Alpha diversity indices. **c** Cladogram of LEfSe analysis showing overrepresented microbial populations. Taxa were significant when assessed by LeFSe (*P* ≤ 0.05 and LDA cutoff > 3.0). **d** Microbial functional predictions revealed the most differentially regulated metabolic pathways in the fecal microbiome at KEGG levels 3 (i.e. Environmental Information Processing)

The NMDS plot revealed a clear separation at the beta diversity level between M-eff and L-eff heifers at d 14 (*P* = 0.05) and d 42 (*P* = 0.01) (Additional file 1: Figure S2). However, no difference was detected between M-eff and L-eff heifers at the beta diversity level during the entire preweaning period (*P* = 0.55) (Fig. 4a and Additional file 1: Figure S3). These results were further confirmed by the lack of differences via Shannon (*P* = 0.50), Chao 1 (*P* = 0.33) and observed species (*P* = 0.33) diversity indices at any tested time-point (Fig. 4b). In addition, alpha diversity indices did not reveal interactions of RFI divergence and time (*P* > 0.05) (Fig. 4b). The LeFSe analysis of microbial taxa (Fig. 4c and Table 4) revealed shifts in the postnatal microbiome communities in response to RFI divergence. For example, M-eff heifers had greater abundance (*P* ≤ 0.05 and LDA cutoff > 2.0) of *Olsenella*, Coriobacteriaceae, *Bacteroides*, Bacteroidaceae, Eubacteriaceae, Clostridiales, *Blautia*, *Dorea*, *GCA-900066575*, *Lachnospiraceae NK3A20*, *Oribacterium*, *Syntrophococcus*, *Ruminococcus*, Lachnospiraceae, *Butyricicoccus*, *Faecalibacterium*, *Negativibacillus*, *Acidaminococcus*, Acidaminococcaceae, *Fusobacterium*, Fusobacteriaceae, Fusobacteriales, *Succinivibrio*, Aeromonadales, unculturebacterium, EMP-G18, but lower (*P* ≤ 0.05 and LDA cutoff > 2.0) *Candidatus Soleaferrea*, *Fournierella*, *Treponema*, and Spirochaetales (Fig. 4c and Table 4).

**Table 4:** Relative abundance (%) of the most differentially abundant bacteria highlighted by LeFSe analysis (*P* ≤ 0.05 and LDA cutoff > 2.0) in feces during the preweaning period in least-efficient (L-eff, *n* = 13) or most-efficient (M-eff, *n* = 13) heifer calves

Bacteria	L-eff	M-eff
g_*Olsenella*	0.144^b^	0.217^a^
f_Coriobacteriaceae	0.008^b^	0.125^a^
g_*Bacteroides*	0.052^b^	0.075^a^
f_Bacteroidaceae	0.022^b^	0.069^a^
f_Eubacteriaceae	0.043^b^	0.310^a^
o_Clostridiales	0.004^b^	0.705^a^
g_*Blautia*	0.901^b^	1.347^a^
g_*Dorea*	0.005^b^	0.068^a^
g_*GCA-900,066,575*	0.016^b^	0.071^a^
g_*Lachnospiraceae NK3A20*	0.011^b^	0.048^a^
g_*Oribacterium*	0.024^b^	0.651^a^
g_*Syntrophococcus*	0.003^b^	0.014^a^
g_*Ruminococcus*	0.026^b^	0.033^a^
f_Lachnospiraceae	0.016^b^	0.021^a^
g_*Butyricicoccus*	0.010^b^	0.019^a^
g_*Candidatus Soleaferrea*	0.017^a^	0.000^b^
g_*Faecalibacterium*	0.154^b^	0.179^a^
g_*Fournierella*	0.011^a^	0.000^b^
g_*Negativibacillus*	0.043^b^	0.075^a^
g_*Acidaminococcus*	0.000^b^	0.069^a^
f_Acidaminococcaceae	0.012^b^	0.024^a^
g_*Fusobacterium*	0.167^b^	0.230^a^
f_Fusobacteriaceae	0.201^b^	0.443^a^
o_Fusobacteriales	0.518^b^	0.590^a^
g_*Succinivibrio*	0.012^b^	0.020^a^
o_Aeromonadales	0.005^b^	0.011^a^
g_*Treponema*	0.023^a^	0.017^b^
f_Spirochaetales	0.052^a^	0.063^b^
f_uncultured bacterium	0.000^b^	0.010^a^
f_uncultured bacterium	0.004^b^	0.027^a^
o_EMP-G18	0.001^b^	0.015^a^

^a,b^Different letters indicate significant differences due to the main maternal effect (*P* < 0.05)

*o* order, *f* family, *g* genus

The M-eff microbiome had greater numbers of functional genes (*P* ≤ 0.05 and LDA cutoff > 2.0) involved in bisphenol degradation, linoleic acid metabolism, naphthalene degradation, primary bile acid biosynthesis, chloroalkane and chloroalkene degradation, transcription related proteins, tyrosine metabolism, glycolysis/gluconeogenesis, secondary bile acid biosynthesis, phenylpropanoid biosynthesis, selenocompound metabolism, bacterial toxins, and styrene degradation (Fig. 4d). In contrast, M-eff microbiome had lower number of functional genes (*P* ≤ 0.05 and LDA cutoff > 2.0) for novobiocin biosynthesis, glycine, serine and threonine metabolism, pantothenate and CoA biosynthesis, C5-branched dibasic acid metabolism, vitamin B_6_ metabolism, amino acid related enzymes, glycan biosynthesis and metabolism, valine, leucine and isoleucine biosynthesis, proteasome, translation proteins, protein processing in endoplasmic reticulum, and *D*-arginine and *D*-ornithine metabolism (Fig. 4d).

### Hindgut metabolome at birth

**Fig. 5:**
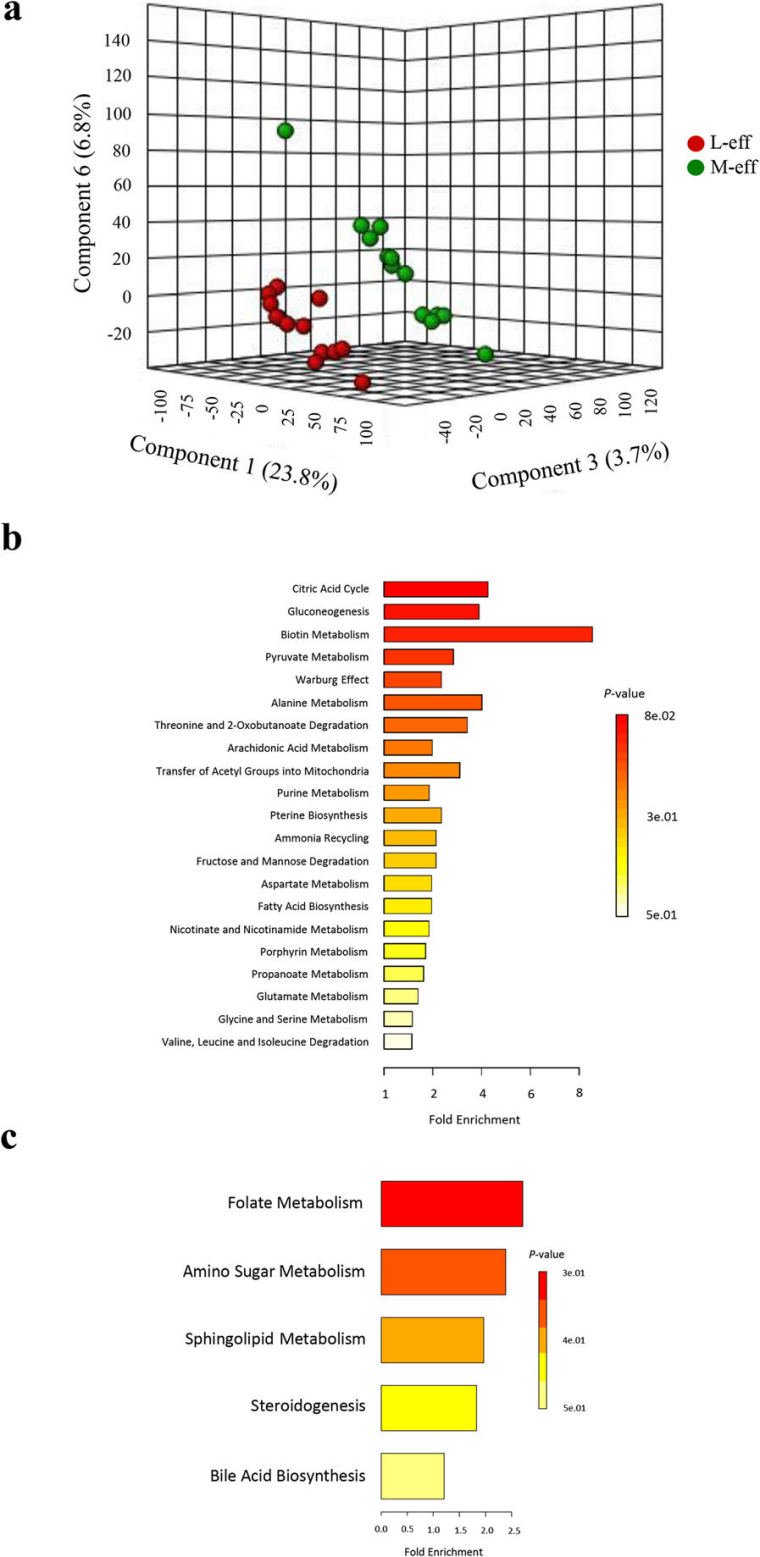
Hindgut metabolome at birth in least-efficient (L-eff, *n* = 13) or most-efficient (M-eff, *n* = 13) heifer calves. **a** 3D scores plot of the partial least square discriminant analysis (PLS-DA) model. **b** and **c** Upregulated and downregulated metabolic pathways in M-eff heifer calves at birth

The PLS-DA plot (Fig. 5a) of metabolomics data revealed a clear separation between M-eff and L-eff newborn heifers at birth. A total of 30 differentially abundant metabolites were identified and annotated (Additional file 1: Figure S4, Additional file 1: Figure S5, and Additional file 2: Table S2). The upregulated metabolites in M-eff (Additional file 1: Figure S4, and Additional file 2: Table S2) were associated with induction (*P* ≤ 0.05) of multiple pathways (Fig. 5b) including citric acid cycle, biotin metabolism, amino acid metabolism, arachidonic acid metabolism, transfer of acetyl groups into mitochondria, purine metabolism, carbohydrate metabolism, and fatty acid biosynthesis. Whereas, downregulated metabolites in M-eff (Additional file 1: Figure S5, and Additional file 2: Table S2) were associated with inhibition (*P* ≤ 0.05) of pathways (Fig. 5c) such as folate metabolism, amino sugar metabolism, sphingolipid metabolism, steroidogenesis, and bile acid biosynthesis.

### Hindgut metabolome during the preweaning period

**Fig. 6:**
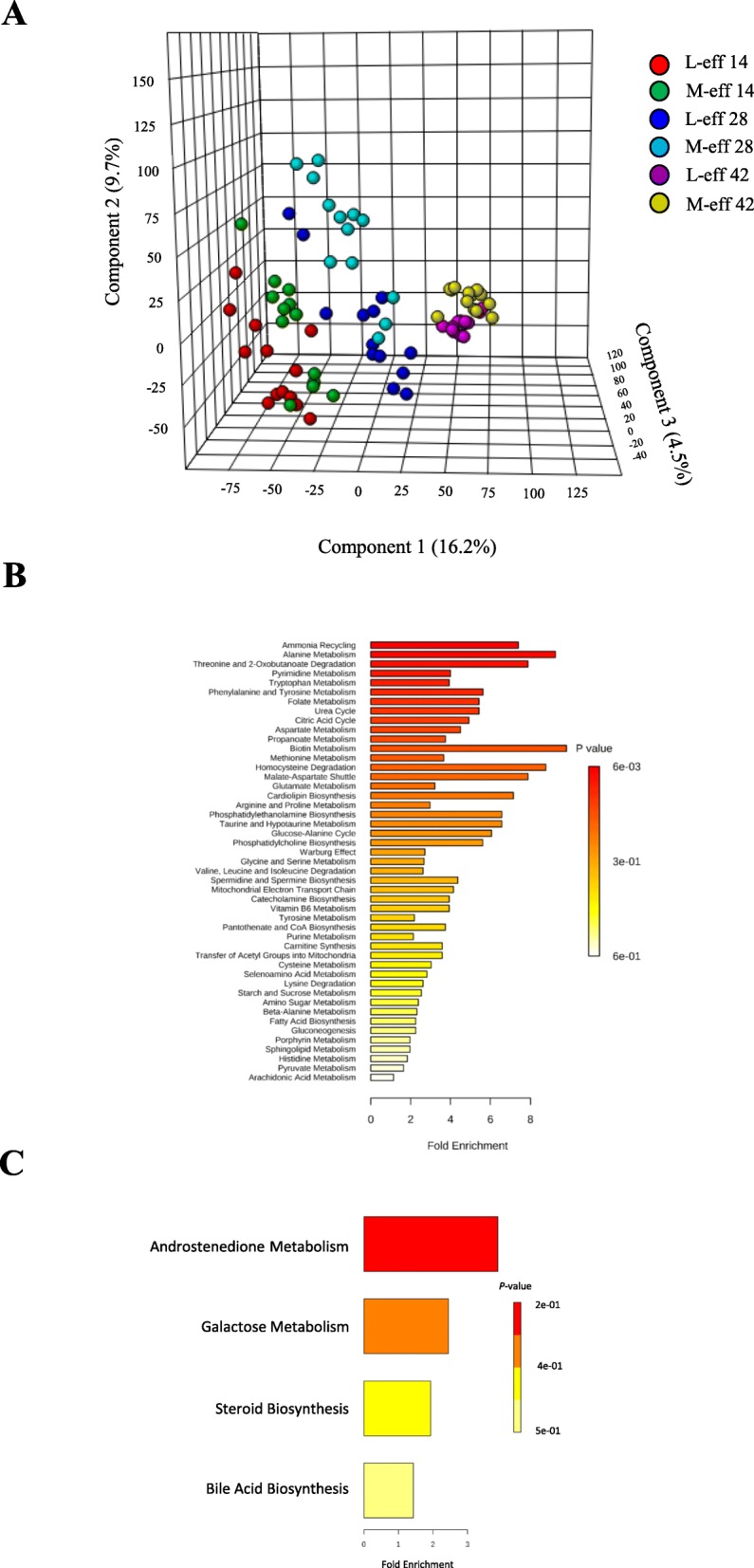
Hindgut metabolome profiles during the preweaning period in least-efficient (L-eff, *n* = 13) or most-efficient (M-eff, *n* = 13) heifer calves. **a** 3D scores plot of partial least square discriminant analysis (PLS-DA). **b** and **c** Upregulated and downregulated metabolic pathways in M-eff heifer calves at birth

The PLS-DA plots (Fig. 6a, and Additional file 1: Figure S6) underscored a clear separation in hindgut metabolite profiles between M-eff and L-eff neonatal heifers during the preweaning period. A total of 30 differentially abundant metabolites were identified and annotated (Additional file 1: Figure S7, Additional file 1: Figure S8, and Additional file 2: Table S3). The upregulated metabolites in M-eff (Additional file 1: Figure S7, and Additional file 2: Table S3) induced (*P* ≤ 0.05) of multiple biological pathways (Fig. 6b) including several associated with nitrogen and amino acid metabolism, energy metabolism, lipid metabolism, purine metabolism, and water-soluble vitamin metabolism. Whereas, downregulated metabolites in M-eff (Additional file 1: Figure S8 and Additional file 2: Table S3) (*P* ≤ 0.05) included pathways (Fig. 6c) such as androstenedione metabolism, galactose metabolism, steroid biosynthesis, and bile acid biosynthesis.

## Discussion

### Growth performance and development

Previous studies with dairy heifers revealed that RFI divergence between postweaned growing heifers (i.e., M-eff vs. L-eff heifers) is maintained during the first lactation [6, 23]. Thus, understanding the biologic differences between M-eff and L-eff heifer calves during the preweaning period is warranted. The present study revealed that during the preweaning period M-eff heifers consumed 54.6% less starter grain DM from birth to weaning (cumulative intake, 10.90 ± 1.66 kg vs. 19.98 ± 1.66 kg), while maintaining similar growth performance and development compared with L-eff calves. These results are in line with other studies in dairy calves indicating that the RFI trait is independent of growth performance [24, 25].

### Hindgut microbiome and metabolome at birth

#### Energy supply

The present study demonstrated that M-eff calves had greater enrichment of key metabolites involved in energy-generating pathways such as citric acid cycle, gluconeogenesis, biotin metabolism, pyruvate metabolism, fructose and mannose degradation, and nicotinate and nicotinamide metabolism [26–28], potentially enhancing the supply of energy to the calf. Furthermore, the induction of metabolic pathways for amino acid (alanine metabolism), vitamin (biotin metabolism) and fatty acid (arachidonic acid metabolism) metabolism at birth in M-eff calves also could have benefitted hindgut development and function during the preweaning period [29]. These data suggest that the metabolic capacity of the hindgut microbiome at birth is important in the context of feed efficiency in early life. Although the present study cannot discern whether the host would have used the metabolites identified, it can be envisioned, for example, that availability of essential nutrients to colonocytes when the “ideal” metabolome profile is established is one factor allowing the animal to achieve optimal rates of growth per unit of feed consumed.

#### Pathogenic bacteria

The fact that hindgut in M-eff heifers at birth had lower abundance of pathogenic bacteria such as *Odoribacter*, Cyanobacteria, *Ruminiclostridium 9*, Prevotellaceae_UCG-001, and *Eubacterium nodatum* typically associated with several diseases, is surprising. For example, *Odoribacter* and Cyanobacteria are positively associated with stress in mice and pigs [30, 31]. *Ruminiclostridium 9* increased in obese rodents [32]. Prevotellaceae_UCG-001 is a well-known bacteria degrading mucus oligosaccharides in the intestine, leading to a smaller mucin layer and the onset of intestinal inflammation in rodents [33]. Previous studies have also reported that *Eubacterium nodatum* is enriched in oral inflammation [34]. Together, the potential increase in nutrient supply to colonocytes and the decrease in number of harmful bacteria in the hindgut of M-eff newborn heifers could have resulted in better hindgut functionality.

### Hindgut microbiome and metabolome during the preweaning period

#### Energy supply

The greater abundance of carbohydrate-fermenting bacteria (i.e.*, Fusobacteria, Blautia,* Lachnospiraceae*, Proteobacteria,* and *Bacteroides*) during the preweaning period in M-eff heifers suggests a better capacity for utilizing complex carbohydrates reaching the hindgut including cellulose, hemicellulose, resistant starch, and xylan [35–37]. This result is supported by previous studies in which digestibility of dry matter, organic matter, fiber, protein, and total digestible nutrients was greater in M-eff heifers [38]. These bacteria can also enhance colonocyte growth and function through the production of VFA. For instance, Fusobacteria, *Faecalibacterium*, *Blautia*, Lachnospiraceae, and *Butyricicoccus* are butyrate-producing bacteria [39–41]. Butyrate is the major energy substrate for colonocytes, and provides many benefits to heifer calves such as improved epithelial tight junctions and reduced inflammatory status [42]. *Blautia* and *Succinivibrio* produce propionate which competes with methanogens for H_2_ [43], leading to less enteric methane production in M-eff buffalo heifers [44]. Absorption of propionate by colonocytes would provide a key hepatic gluconeogenic precursor to heifers, hence, indirectly increasing the supply of glucose from what is absorbed from the lumen of the small intestine [45].

Although hindgut VFA concentrations were not assessed in the current study, previous data revealed that M-eff adult dairy cows at mid-lactation had greater propionate and propionate:acetate ratio in the rumen [11]. Therefore, we speculate that greater abundance of VFA-producing bacteria in M-eff heifers would not only benefit colonocytes but also liver metabolism. The greater number of altered microbial genes across metabolic pathways involved in energy metabolism including citric acid cycle [46], pyruvate metabolism [47], glycolysis/gluconeogenesis [48], malate-aspartate shuttle [49], transfer of acetyl groups into mitochondria [50], mitochondrial electron transport chain [51], and fatty acid biosynthesis [52] in M-eff heifers underscored the enhanced metabolic capacity of the microbiome. Measurement of hindgut VFA should be performed in future RFI studies with dairy calves.

#### Vitamin supply

Our results revealed that M-eff heifers upregulated the metabolism of important vitamins such as biotin (vitamin B_7_), vitamin B_6_, and folate (vitamin B_9_). Cattle cannot synthesize biotin, therefore, rely on ruminal and hindgut bacteria [53]. Biotin is an important cofactor for metabolic reactions such as glucose, amino acid and fatty acid metabolism [54]. Biotin also regulates important functions such as cell signaling and mucosal immune responses [55]. Through the upregulation of one-carbon metabolism, neurotransmitter biosynthesis (dopamine and serotonin) and oxidative stress reduction [56, 57], greater production of vitamin B_6_ and folate (vitamin B_9_) could become key cofactors for regulation of cellular metabolism [58, 59]. Together, data suggest that induction of B vitamins metabolism in the microbiome of M-eff heifers might have contributed to increasing the supply of B vitamins during the preweaning period.

#### Amino acid supply

Most dietary amino acids (AA) are absorbed in the small intestine, but substantial amounts can reach the hindgut [60]. The prevailing notion is that mammals, including ruminants, do not absorb AA from the hindgut [61]. However, a series of studies suggest the opposite. For example, early studies using infusions of ^15^N-labeled lysine and ^14^C-labeled isoleucine into the cecum of growing pigs revealed their absorption from the hindgut [62, 63]. The detection of ^15^N-labelled AA in blood of pigs and ponies infused with ^15^N-labeled microbes into the cecum indicated colonocytes can absorb microbial-derived AA [64, 65]. The absorption of AA from the intestinal lumen requires a large family of AA transporters, many of which are expressed in the hindgut of humans, pigs and rodents including neutral and basic amino acid transporters (SLC6A14, SLC3A1) and *L*-type amino acid transporters (SLC7A5, SLC7A6) [60]. Detection of these AA transporters in the hindgut of neonatal calves should provide additional support for the notion that AA absorption occurs in the hindgut. Although the potential availability of these important AA for colonocyte absorption is suggested, hindgut bacteria could also metabolize them further. For example, uptake of AA such as glutamate and tryptophan by *Peptostreptococcus* bacteria in the human hindgut [66], likely leads to reduced availability to colonocytes. Future studies are warranted to unmask the relationships between microbiome and AA availability in cattle hindgut.

Among the AA-related pathways enriched in M-eff heifers, arginine and proline are noteworthy because of their involvement in RNA synthesis and protein glycosylation both of which are essential for cellular function [67]. Methionine is required for spermidine and spermine biosynthesis [68], compounds that help alleviate oxidative stress [69]. The upregulation of spermidine and spermine biosynthesis observed in fecal metabolome of M-eff heifers agrees with the induction of methionine metabolism. Tyrosine and its metabolites such as cinnamic acids and p-hydroxyphenylacetic reduce reactive oxygen species (ROS) production [70]. Assuming that some of these metabolites would be available for uptake by intestinal cells, the upregulation of spermidine, spermine and tyrosine metabolism in M-eff heifers could exert a positive effect in the context of reducing oxidant status, potentially contributing to enhanced hindgut integrity.

Tyrosine, tryptophan, and phenylalanine are required for serotonin and dopamine synthesis, both of which are important neurotransmitters [71]. The induction of tyrosine, tryptophan, and phenylalanine metabolism in M-eff heifers suggested a potential line of communication between hindgut and brain during the preweaning period. The degradation of the branched-chain amino acids (BCAA) valine, leucine, and isoleucine generates succinyl-CoA and acetyl-CoA, both of which could contribute to energy synthesis via the upregulation of the citric acid cycle [72]. Furthermore, the degradation of BCAA produces α-keto acids, which can induce cellular growth through the activation of mechanistic target of rapamycin (mTOR) signaling [73]. Therefore, the induction of BCAA catabolism in M-eff heifers represents another adaptation that could increase the availability of metabolically-important compounds for neonatal heifers.

Although some previous RFI studies with dairy cattle have used a limited number of animals (5 to 8 animal/group) to compare between extreme M-eff and L-eff individuals [74, 75], greater sample size clearly could enhance the ability for detecting biological effects in these kinds of experiments [76]. In fact, a recent study with beef cattle argued that increasing the number of animals would improve RFI divergence because DMI is repeatable across different life stages including the growing period, i.e. a period similar to the preweaning stage in calves [77]. Therefore, we used the entire cohort of calves available to us (13 M-eff vs. 13 L-eff) in the current study. Results from the analyses of growth performance, microbiome, and metabolome supports our strategy. For example, the Shapiro-Wilks test for normality of body measurements and growth performance at birth and during the preweaning period, respectively, revealed a *P*-value that was > 0.05 for the 26 calves at each individual time-point (data not shown), confirming the data were normally distributed, with no odd values (i.e. values greater than 95% confidence interval between M-eff and L-eff calves) including those calves whose RFI coefficient was within the range of experimental error for RFI divergence. Furthermore, microbiome and metabolome analyses indicated a clear separation between M-eff and L-eff calves at each individual time-point. We did not detect an overlap between M-eff and L-eff groups from birth to weaning, supporting the use of the entire cohort of calves for evaluating M-eff and L-eff. More studies are warranted to validate the use of full animal cohorts in RFI studies.

## Conclusions

The divergence in RFI during early life is associated with unique microbiome-metabolome profiles in the hindgut of dairy calves. The beneficial profiles in hindgut microbiome and metabolome at birth before colostrum feeding shape the early hindgut microbiome and might partly determine superior feed efficiency. That idea is supported by the similar growth and body development in the more-efficient calves that consumed less starter DMI than least-efficient calves during the preweaning period. The present study could not discern whether M-eff heifer calves absorbed and utilized the differentially expressed metabolites identified in the hindgut. However, in spite of consuming less solid feed and similar amounts of milk replacer during the preweaning period, it can be envisioned that greater availability of essential vitamins and amino acids to colonocytes might support optimal growth rates in M-eff compared with L-eff calves. Whether the microbiome-metabolome profiles at birth denote dam-to-fetus efflux of commensal bacteria during pregnancy remains to be determined.

## Supplementary information

**Additional file 1: Figure S1.** Phyla level taxonomic distribution in hindgut samples at birth in most-efficient (M-eff) and least-efficient (L-eff) heifer calves. **Figure S2.** Non-metric multidimensional scaling (NMDS) plot of fecal microbiome profiles during the preweaning period in least-efficient (L-eff, *n* = 13) or most-efficient (M-eff, *n* = 13) heifer calves at (**A**) day 14, (**B**) day 28, and (**C**) day 42 of age. **Figure S3.** Phyla level taxonomic distribution in hindgut samples during the preweaning period at day 14, 28 and 42 of age in most-efficient (M-eff) and least-efficient (L-eff) heifer calves. **Figure S4.** Upregulated hindgut metabolites in M-eff heifer calves at birth strongly influencing metabolome discrimination between most-efficient (M-eff, *n* = 13) and least-efficient (L-eff, *n* = 13) heifer calves assessed by partial least square discriminant analysis (PLS-DA)l. **Figure S5.** Downregulated hindgut metabolites in M-eff heifer calves at birth strongly influencing metabolome discrimination between most-efficient (M-eff, *n* = 13) and least-efficient (L-eff, *n* = 13) heifer calves assessed by partial least square discriminant analysis (PLS-DA). **Figure S6.** Scores plot of partial least square discriminant analysis (PLS-DA) for hindgut metabolome profiles during the preweaning period in least-efficient (L-eff, *n* = 13) or most-efficient (M-eff, *n* = 13) heifer calves at (**A**) day 14, (**B**) day 28, and (**C**) day 42 of age. **Figure S7.** Upregulated hindgut metabolites in M-eff heifer calves during the preweaning period strongly influencing metabolome discrimination between most-efficient (M-eff, *n* = 13) and least-efficient (L-eff, *n* = 13) heifer calves assessed by partial least square discriminant analysis (PLS-DA). **Figure S8.** Downregulated hindgut metabolites in M-eff heifer calves during the preweaning period strongly influencing metabolome discrimination between most-efficient (M-eff, *n* = 13) and least-efficient (L-eff, *n* = 13) heifer calves assessed by partial least square discriminant analysis (PLS-DA)

**Additional file 2: Table S1.** Nutrient composition and amino acid profiles (mean ± standard deviation) of milk replacer (Advance Excelerate, Milk Specialties, Carpentersville, IL, USA) and starter grain (Ampli-Calf Starter 20; Purina Animal Nutrition, Shoreview, MN, USA) fed during the preweaning period to the most-efficient (M-eff, *n* = 13) or least-efficient (L-eff, *n* = 13) heifer calves. **Table S2.** Chemical taxonomy of top metabolites strongly influencing discrimination assessed by partial least squares discriminate analysis (PLS-DA) that were upregulated and downregulated in the hindgut of most-efficient (M-eff, *n* = 13) heifer calves compared with least-efficient (L-eff, *n* = 13) heifer calves at birth, following the conditions of VIP > 1.0 and |p-(corr)|.

## Data Availability

The datasets during and/or analyzed during the current study available from the corresponding authors on reasonable request.

## Abbreviations

ADG: Average daily gain
ASVs: Amplicon sequence variants
BW: Body weight
BWG: Cumulative body weight gain
CP: Crude protein
DMI: Dry matter intake
HMDB: Human metabolome database
KEGG: Kyoto encyclopedia of genes and genomes
LDA: Linear discriminant analysis
L-eff: Least feed-efficient heifer calves
LEfSe: Linear discriminant analysis Effect Size
MBW: Metabolic body weight
MBW: Mid-test metabolic body weight
M-eff: Most feed-efficient heifer calves
MW: Molecular weight
NDF: Neutral detergent fiber
NMDS: Non-metric multidimensional scaling
PERMANOVA: Permutational multivariate analysis of variance
PICRUSt: Phylogenetic investigation of communities by reconstruction of unobserved states
PLS-DA: Partial least squares discriminant analysis
QIIME: Quantitative insights into microbial ecology
RFI: Residual feed intake
VFA: Volatile fatty acids
VIP: Variable importance in the projection
