# Supplementary figures and images for: Residual feed intake divergence during the preweaning period is associated with unique hindgut microbiome and metabolome profiles in neonatal Holstein heifer calves
Source: J Anim Sci Biotechnol. 2020 Jan 20;11:13. doi: 10.1186/s40104-019-0406-x (PMC6972010; doi:10.1186/s40104-019-0406-x)

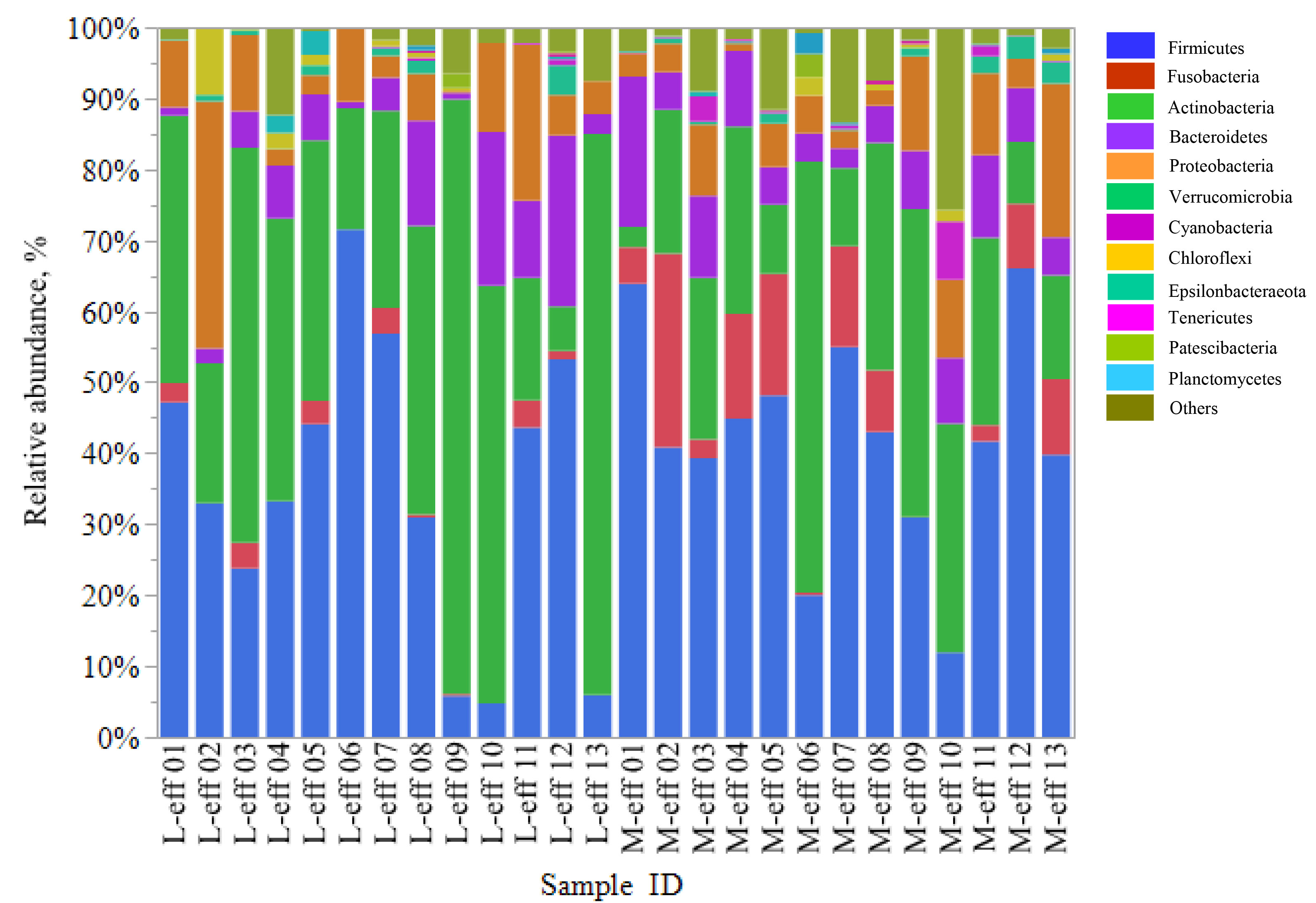

Supplement: Supplementary file 1 — Additional file 1: Figure S1. Phyla level taxonomic distribution in hindgut samples at birth in most-efficient (M-eff) and least-efficient (L-eff) heifer calves. Figure S2. Non-metric multidimensional scaling (NMDS) plot of fecal microbiome profiles during the preweaning period in least-efficient (L-eff, n = 13) or most-efficient (M-eff, n = 13) heifer calves at (A) day 14, (B) day 28, and (C) day 42 of age. Figure S3. Phyla level taxonomic distribution in hindgut samples during the preweaning period at day 14, 28 and 42 of age in most-efficient (M-eff) and least-efficient (L-eff) heifer calves. Figure S4. Upregulated hindgut metabolites in M-eff heifer calves at birth strongly influencing metabolome discrimination between most-efficient (M-eff, n = 13) and least-efficient (L-eff, n = 13) heifer calves assessed by partial least square discriminant analysis (PLS-DA)l. Figure S5. Downregulated hindgut metabolites in M-eff heifer calves at birth strongly influencing metabolome discrimination between most-efficient (M-eff, n = 13) and least-efficient (L-eff, n = 13) heifer calves assessed by partial least square discriminant analysis (PLS-DA). Figure S6. Scores plot of partial least square discriminant analysis (PLS-DA) for hindgut metabolome profiles during the preweaning period in least-efficient (L-eff, n = 13) or most-efficient (M-eff, n = 13) heifer calves at (A) day 14, (B) day 28, and (C) day 42 of age. Figure S7. Upregulated hindgut metabolites in M-eff heifer calves during the preweaning period strongly influencing metabolome discrimination between most-efficient (M-eff, n = 13) and least-efficient (L-eff, n = 13) heifer calves assessed by partial least square discriminant analysis (PLS-DA). Figure S8. Downregulated hindgut metabolites in M-eff heifer calves during the preweaning period strongly influencing metabolome discrimination between most-efficient (M-eff, n = 13) and least-efficient (L-eff, n = 13) heifer calves assessed by partial least square discrimi [file 40104_2019_406_MOESM1_ESM.zip › Additional file 1 Fig. S1.jpg]

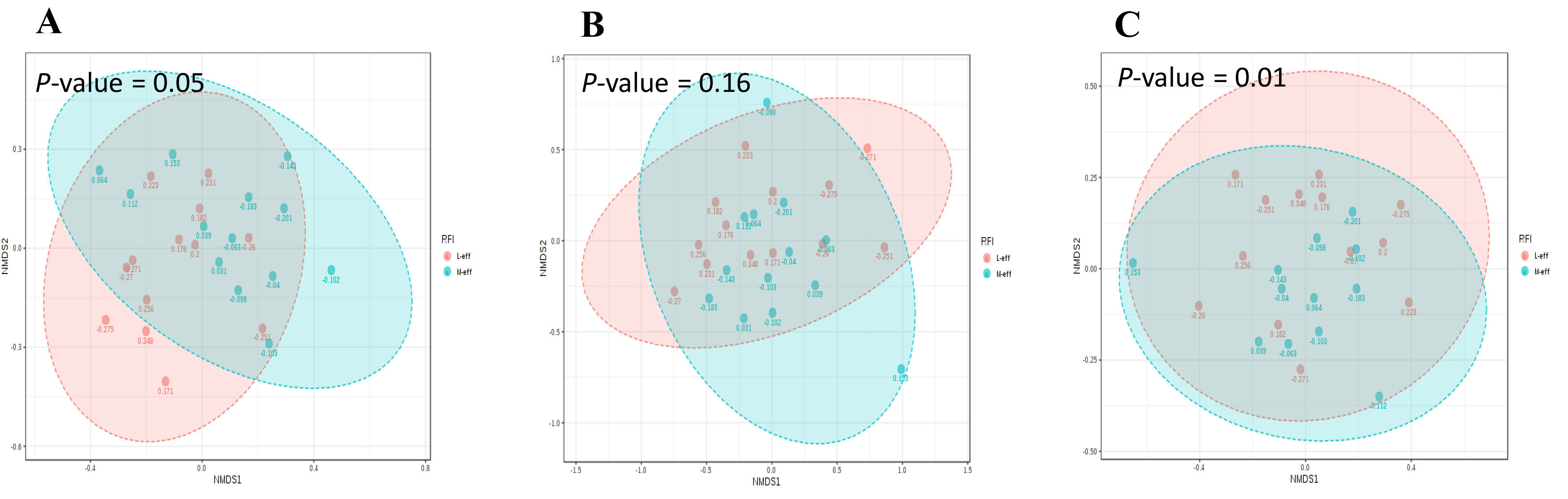

Supplement: Supplementary file 1 — Additional file 1: Figure S1. Phyla level taxonomic distribution in hindgut samples at birth in most-efficient (M-eff) and least-efficient (L-eff) heifer calves. Figure S2. Non-metric multidimensional scaling (NMDS) plot of fecal microbiome profiles during the preweaning period in least-efficient (L-eff, n = 13) or most-efficient (M-eff, n = 13) heifer calves at (A) day 14, (B) day 28, and (C) day 42 of age. Figure S3. Phyla level taxonomic distribution in hindgut samples during the preweaning period at day 14, 28 and 42 of age in most-efficient (M-eff) and least-efficient (L-eff) heifer calves. Figure S4. Upregulated hindgut metabolites in M-eff heifer calves at birth strongly influencing metabolome discrimination between most-efficient (M-eff, n = 13) and least-efficient (L-eff, n = 13) heifer calves assessed by partial least square discriminant analysis (PLS-DA)l. Figure S5. Downregulated hindgut metabolites in M-eff heifer calves at birth strongly influencing metabolome discrimination between most-efficient (M-eff, n = 13) and least-efficient (L-eff, n = 13) heifer calves assessed by partial least square discriminant analysis (PLS-DA). Figure S6. Scores plot of partial least square discriminant analysis (PLS-DA) for hindgut metabolome profiles during the preweaning period in least-efficient (L-eff, n = 13) or most-efficient (M-eff, n = 13) heifer calves at (A) day 14, (B) day 28, and (C) day 42 of age. Figure S7. Upregulated hindgut metabolites in M-eff heifer calves during the preweaning period strongly influencing metabolome discrimination between most-efficient (M-eff, n = 13) and least-efficient (L-eff, n = 13) heifer calves assessed by partial least square discriminant analysis (PLS-DA). Figure S8. Downregulated hindgut metabolites in M-eff heifer calves during the preweaning period strongly influencing metabolome discrimination between most-efficient (M-eff, n = 13) and least-efficient (L-eff, n = 13) heifer calves assessed by partial least square discrimi [file 40104_2019_406_MOESM1_ESM.zip › Additional file 1 Fig. S2.jpg]

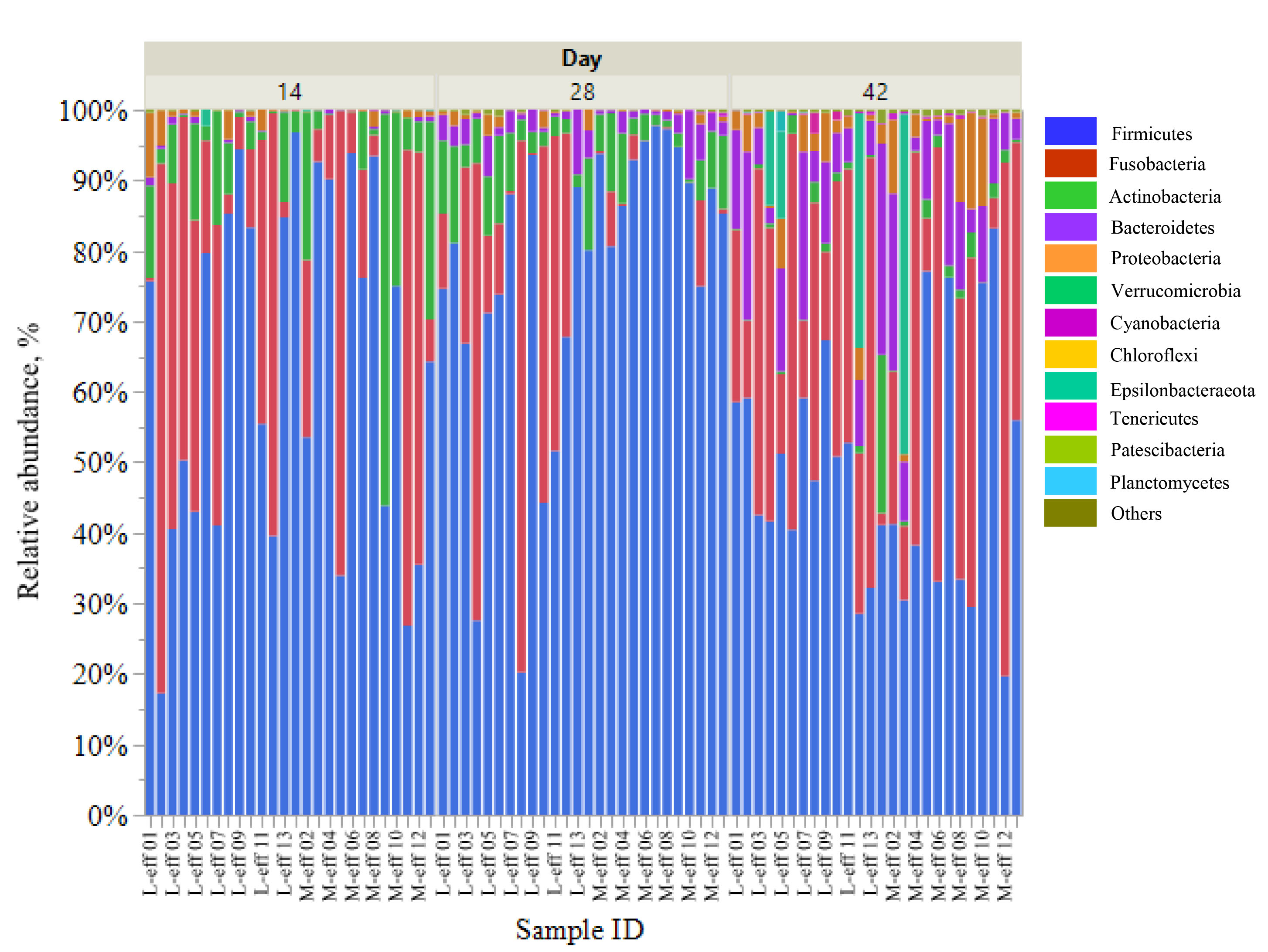

Supplement: Supplementary file 1 — Additional file 1: Figure S1. Phyla level taxonomic distribution in hindgut samples at birth in most-efficient (M-eff) and least-efficient (L-eff) heifer calves. Figure S2. Non-metric multidimensional scaling (NMDS) plot of fecal microbiome profiles during the preweaning period in least-efficient (L-eff, n = 13) or most-efficient (M-eff, n = 13) heifer calves at (A) day 14, (B) day 28, and (C) day 42 of age. Figure S3. Phyla level taxonomic distribution in hindgut samples during the preweaning period at day 14, 28 and 42 of age in most-efficient (M-eff) and least-efficient (L-eff) heifer calves. Figure S4. Upregulated hindgut metabolites in M-eff heifer calves at birth strongly influencing metabolome discrimination between most-efficient (M-eff, n = 13) and least-efficient (L-eff, n = 13) heifer calves assessed by partial least square discriminant analysis (PLS-DA)l. Figure S5. Downregulated hindgut metabolites in M-eff heifer calves at birth strongly influencing metabolome discrimination between most-efficient (M-eff, n = 13) and least-efficient (L-eff, n = 13) heifer calves assessed by partial least square discriminant analysis (PLS-DA). Figure S6. Scores plot of partial least square discriminant analysis (PLS-DA) for hindgut metabolome profiles during the preweaning period in least-efficient (L-eff, n = 13) or most-efficient (M-eff, n = 13) heifer calves at (A) day 14, (B) day 28, and (C) day 42 of age. Figure S7. Upregulated hindgut metabolites in M-eff heifer calves during the preweaning period strongly influencing metabolome discrimination between most-efficient (M-eff, n = 13) and least-efficient (L-eff, n = 13) heifer calves assessed by partial least square discriminant analysis (PLS-DA). Figure S8. Downregulated hindgut metabolites in M-eff heifer calves during the preweaning period strongly influencing metabolome discrimination between most-efficient (M-eff, n = 13) and least-efficient (L-eff, n = 13) heifer calves assessed by partial least square discrimi [file 40104_2019_406_MOESM1_ESM.zip › Additional file 1 Fig. S3.jpg]

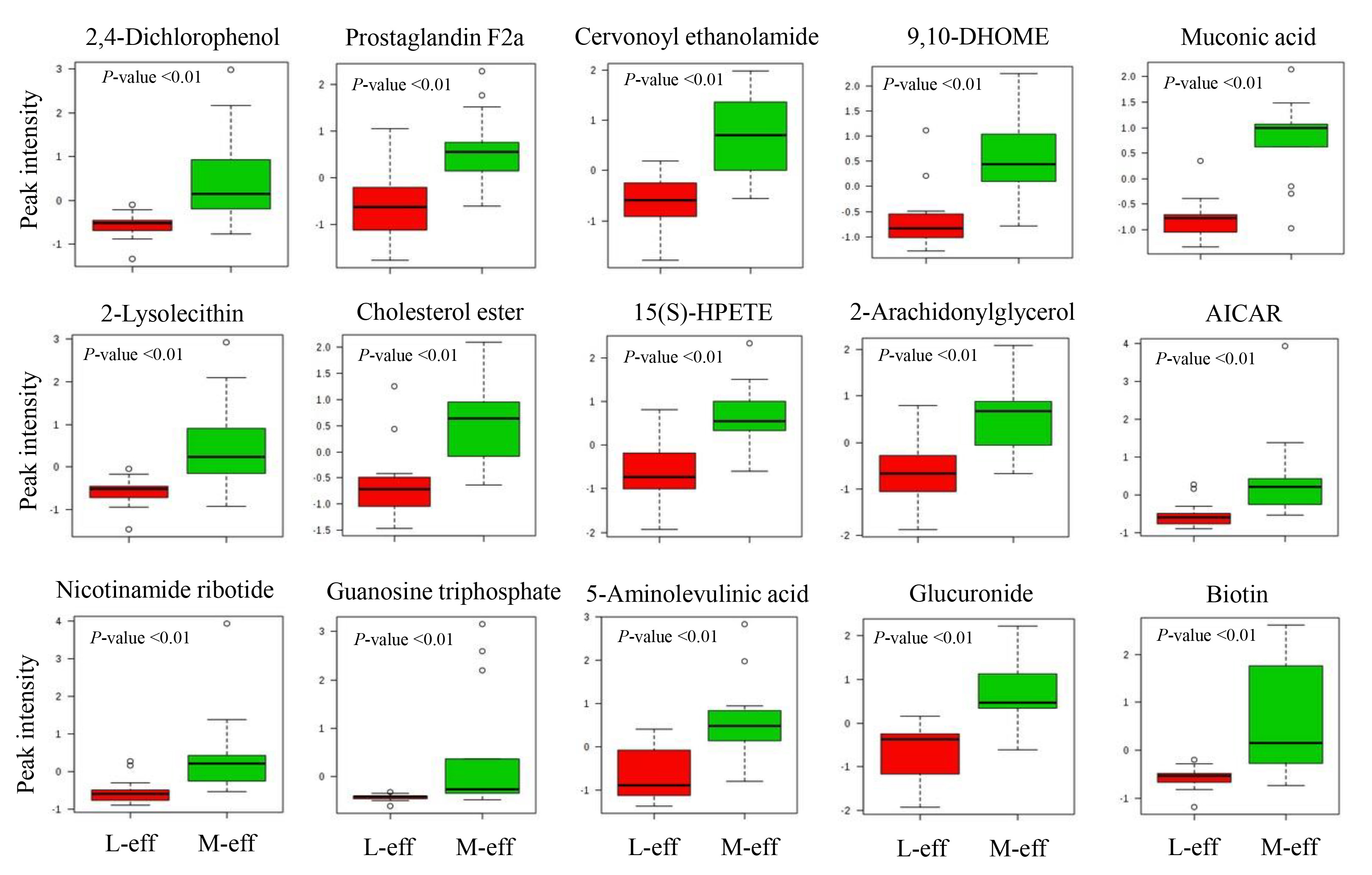

Supplement: Supplementary file 1 — Additional file 1: Figure S1. Phyla level taxonomic distribution in hindgut samples at birth in most-efficient (M-eff) and least-efficient (L-eff) heifer calves. Figure S2. Non-metric multidimensional scaling (NMDS) plot of fecal microbiome profiles during the preweaning period in least-efficient (L-eff, n = 13) or most-efficient (M-eff, n = 13) heifer calves at (A) day 14, (B) day 28, and (C) day 42 of age. Figure S3. Phyla level taxonomic distribution in hindgut samples during the preweaning period at day 14, 28 and 42 of age in most-efficient (M-eff) and least-efficient (L-eff) heifer calves. Figure S4. Upregulated hindgut metabolites in M-eff heifer calves at birth strongly influencing metabolome discrimination between most-efficient (M-eff, n = 13) and least-efficient (L-eff, n = 13) heifer calves assessed by partial least square discriminant analysis (PLS-DA)l. Figure S5. Downregulated hindgut metabolites in M-eff heifer calves at birth strongly influencing metabolome discrimination between most-efficient (M-eff, n = 13) and least-efficient (L-eff, n = 13) heifer calves assessed by partial least square discriminant analysis (PLS-DA). Figure S6. Scores plot of partial least square discriminant analysis (PLS-DA) for hindgut metabolome profiles during the preweaning period in least-efficient (L-eff, n = 13) or most-efficient (M-eff, n = 13) heifer calves at (A) day 14, (B) day 28, and (C) day 42 of age. Figure S7. Upregulated hindgut metabolites in M-eff heifer calves during the preweaning period strongly influencing metabolome discrimination between most-efficient (M-eff, n = 13) and least-efficient (L-eff, n = 13) heifer calves assessed by partial least square discriminant analysis (PLS-DA). Figure S8. Downregulated hindgut metabolites in M-eff heifer calves during the preweaning period strongly influencing metabolome discrimination between most-efficient (M-eff, n = 13) and least-efficient (L-eff, n = 13) heifer calves assessed by partial least square discrimi [file 40104_2019_406_MOESM1_ESM.zip › Additional file 1 Fig. S4.jpg]

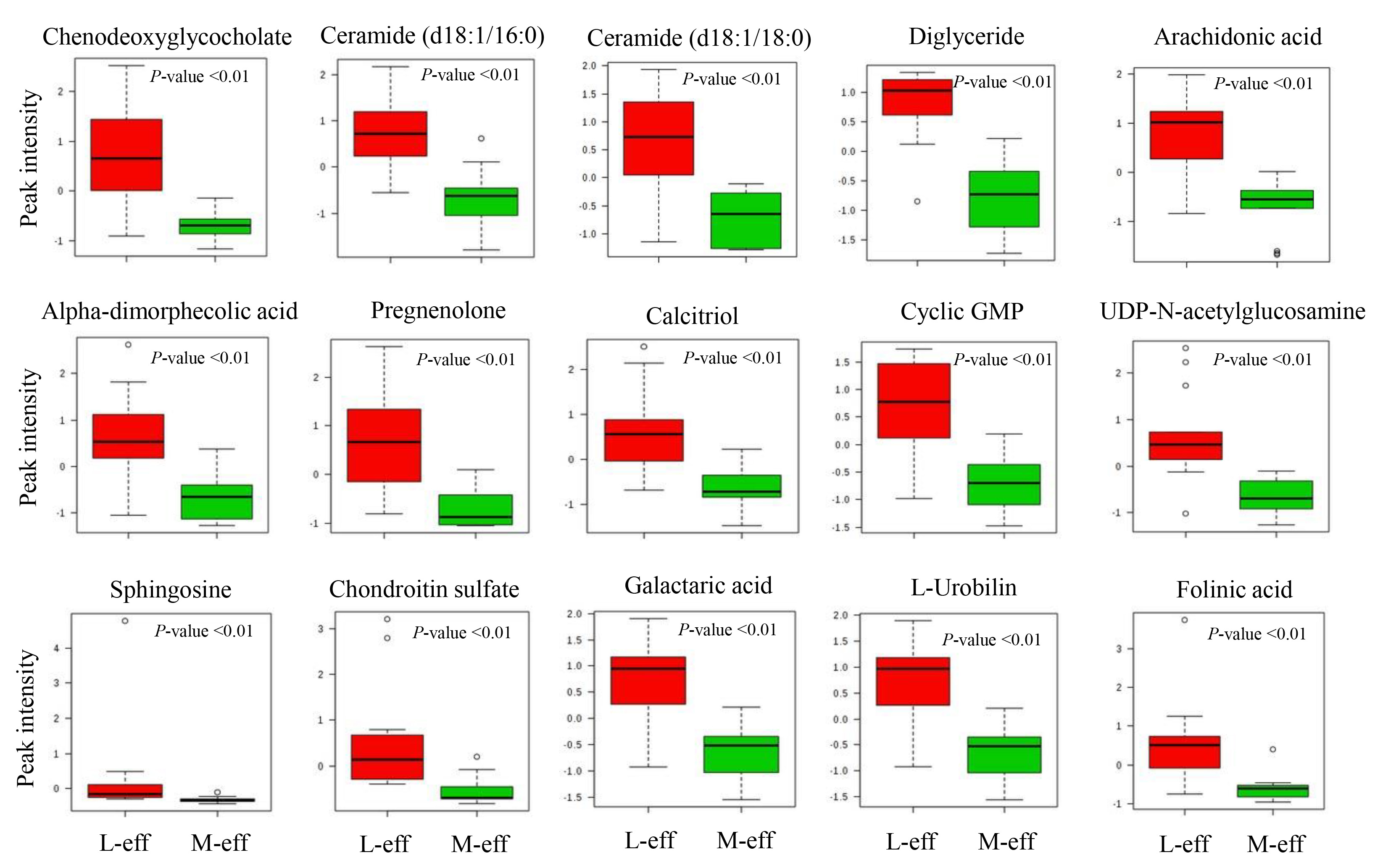

Supplement: Supplementary file 1 — Additional file 1: Figure S1. Phyla level taxonomic distribution in hindgut samples at birth in most-efficient (M-eff) and least-efficient (L-eff) heifer calves. Figure S2. Non-metric multidimensional scaling (NMDS) plot of fecal microbiome profiles during the preweaning period in least-efficient (L-eff, n = 13) or most-efficient (M-eff, n = 13) heifer calves at (A) day 14, (B) day 28, and (C) day 42 of age. Figure S3. Phyla level taxonomic distribution in hindgut samples during the preweaning period at day 14, 28 and 42 of age in most-efficient (M-eff) and least-efficient (L-eff) heifer calves. Figure S4. Upregulated hindgut metabolites in M-eff heifer calves at birth strongly influencing metabolome discrimination between most-efficient (M-eff, n = 13) and least-efficient (L-eff, n = 13) heifer calves assessed by partial least square discriminant analysis (PLS-DA)l. Figure S5. Downregulated hindgut metabolites in M-eff heifer calves at birth strongly influencing metabolome discrimination between most-efficient (M-eff, n = 13) and least-efficient (L-eff, n = 13) heifer calves assessed by partial least square discriminant analysis (PLS-DA). Figure S6. Scores plot of partial least square discriminant analysis (PLS-DA) for hindgut metabolome profiles during the preweaning period in least-efficient (L-eff, n = 13) or most-efficient (M-eff, n = 13) heifer calves at (A) day 14, (B) day 28, and (C) day 42 of age. Figure S7. Upregulated hindgut metabolites in M-eff heifer calves during the preweaning period strongly influencing metabolome discrimination between most-efficient (M-eff, n = 13) and least-efficient (L-eff, n = 13) heifer calves assessed by partial least square discriminant analysis (PLS-DA). Figure S8. Downregulated hindgut metabolites in M-eff heifer calves during the preweaning period strongly influencing metabolome discrimination between most-efficient (M-eff, n = 13) and least-efficient (L-eff, n = 13) heifer calves assessed by partial least square discrimi [file 40104_2019_406_MOESM1_ESM.zip › Additional file 1 Fig. S5.jpg]

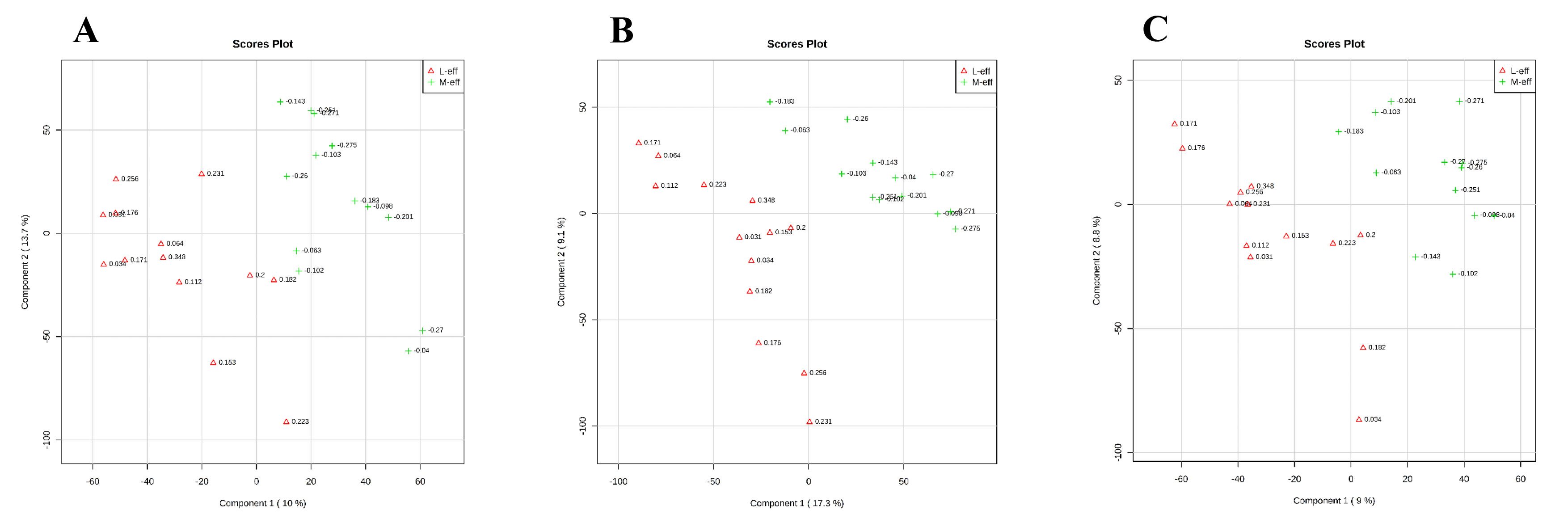

Supplement: Supplementary file 1 — Additional file 1: Figure S1. Phyla level taxonomic distribution in hindgut samples at birth in most-efficient (M-eff) and least-efficient (L-eff) heifer calves. Figure S2. Non-metric multidimensional scaling (NMDS) plot of fecal microbiome profiles during the preweaning period in least-efficient (L-eff, n = 13) or most-efficient (M-eff, n = 13) heifer calves at (A) day 14, (B) day 28, and (C) day 42 of age. Figure S3. Phyla level taxonomic distribution in hindgut samples during the preweaning period at day 14, 28 and 42 of age in most-efficient (M-eff) and least-efficient (L-eff) heifer calves. Figure S4. Upregulated hindgut metabolites in M-eff heifer calves at birth strongly influencing metabolome discrimination between most-efficient (M-eff, n = 13) and least-efficient (L-eff, n = 13) heifer calves assessed by partial least square discriminant analysis (PLS-DA)l. Figure S5. Downregulated hindgut metabolites in M-eff heifer calves at birth strongly influencing metabolome discrimination between most-efficient (M-eff, n = 13) and least-efficient (L-eff, n = 13) heifer calves assessed by partial least square discriminant analysis (PLS-DA). Figure S6. Scores plot of partial least square discriminant analysis (PLS-DA) for hindgut metabolome profiles during the preweaning period in least-efficient (L-eff, n = 13) or most-efficient (M-eff, n = 13) heifer calves at (A) day 14, (B) day 28, and (C) day 42 of age. Figure S7. Upregulated hindgut metabolites in M-eff heifer calves during the preweaning period strongly influencing metabolome discrimination between most-efficient (M-eff, n = 13) and least-efficient (L-eff, n = 13) heifer calves assessed by partial least square discriminant analysis (PLS-DA). Figure S8. Downregulated hindgut metabolites in M-eff heifer calves during the preweaning period strongly influencing metabolome discrimination between most-efficient (M-eff, n = 13) and least-efficient (L-eff, n = 13) heifer calves assessed by partial least square discrimi [file 40104_2019_406_MOESM1_ESM.zip › Additional file 1 Fig. S6.jpg]

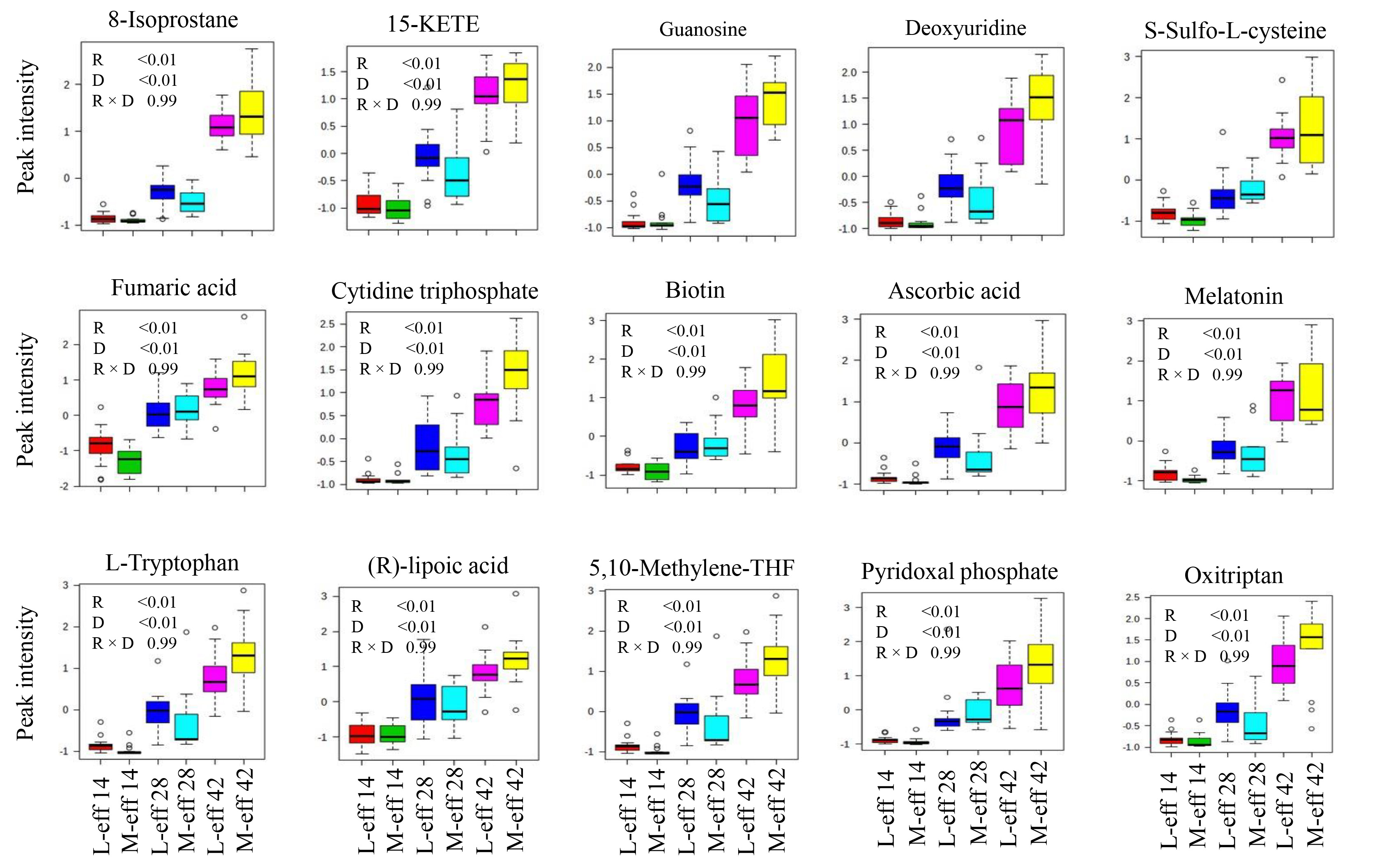

Supplement: Supplementary file 1 — Additional file 1: Figure S1. Phyla level taxonomic distribution in hindgut samples at birth in most-efficient (M-eff) and least-efficient (L-eff) heifer calves. Figure S2. Non-metric multidimensional scaling (NMDS) plot of fecal microbiome profiles during the preweaning period in least-efficient (L-eff, n = 13) or most-efficient (M-eff, n = 13) heifer calves at (A) day 14, (B) day 28, and (C) day 42 of age. Figure S3. Phyla level taxonomic distribution in hindgut samples during the preweaning period at day 14, 28 and 42 of age in most-efficient (M-eff) and least-efficient (L-eff) heifer calves. Figure S4. Upregulated hindgut metabolites in M-eff heifer calves at birth strongly influencing metabolome discrimination between most-efficient (M-eff, n = 13) and least-efficient (L-eff, n = 13) heifer calves assessed by partial least square discriminant analysis (PLS-DA)l. Figure S5. Downregulated hindgut metabolites in M-eff heifer calves at birth strongly influencing metabolome discrimination between most-efficient (M-eff, n = 13) and least-efficient (L-eff, n = 13) heifer calves assessed by partial least square discriminant analysis (PLS-DA). Figure S6. Scores plot of partial least square discriminant analysis (PLS-DA) for hindgut metabolome profiles during the preweaning period in least-efficient (L-eff, n = 13) or most-efficient (M-eff, n = 13) heifer calves at (A) day 14, (B) day 28, and (C) day 42 of age. Figure S7. Upregulated hindgut metabolites in M-eff heifer calves during the preweaning period strongly influencing metabolome discrimination between most-efficient (M-eff, n = 13) and least-efficient (L-eff, n = 13) heifer calves assessed by partial least square discriminant analysis (PLS-DA). Figure S8. Downregulated hindgut metabolites in M-eff heifer calves during the preweaning period strongly influencing metabolome discrimination between most-efficient (M-eff, n = 13) and least-efficient (L-eff, n = 13) heifer calves assessed by partial least square discrimi [file 40104_2019_406_MOESM1_ESM.zip › Additional file 1 Fig. S7.jpg]

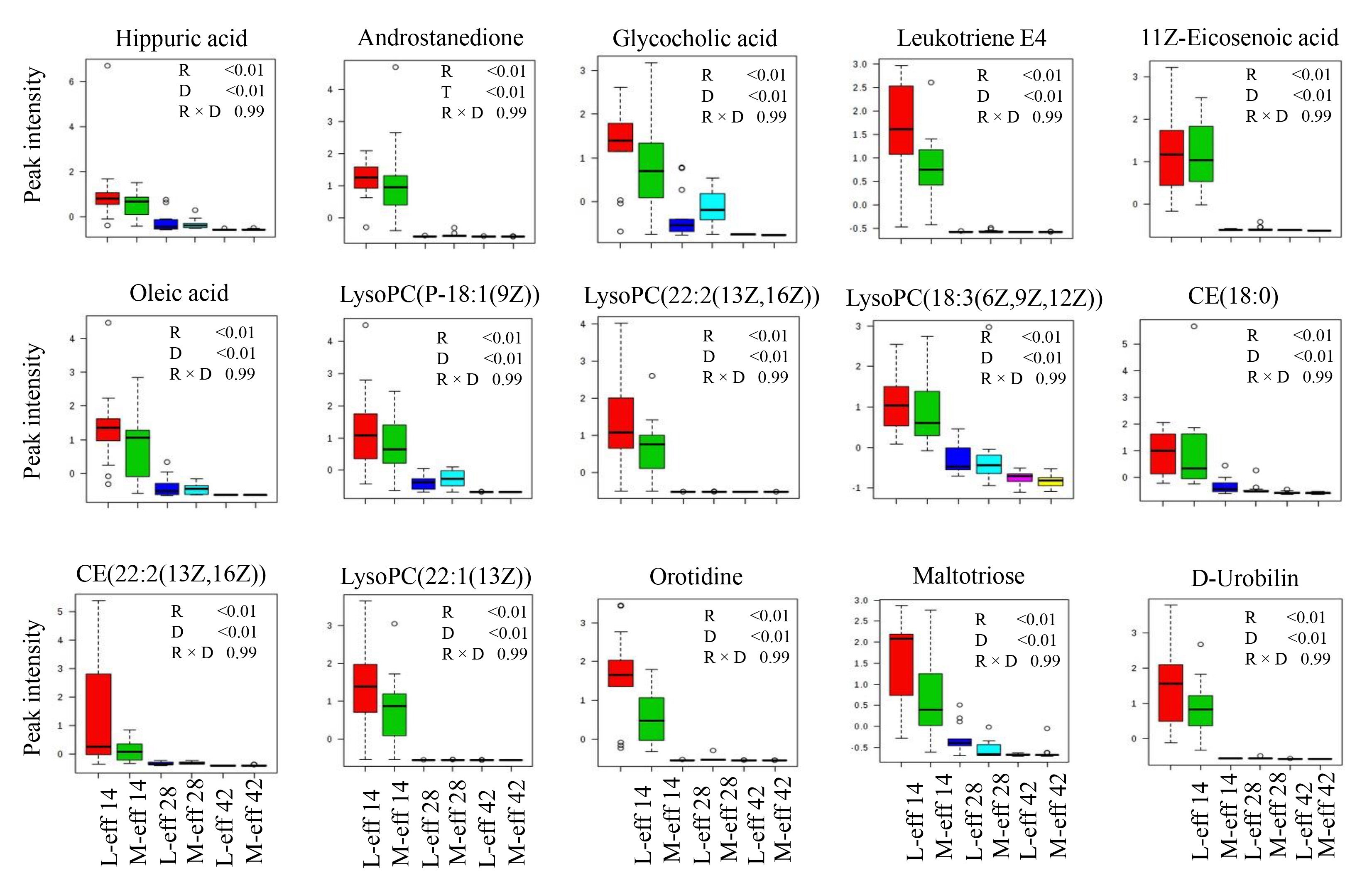

Supplement: Supplementary file 1 — Additional file 1: Figure S1. Phyla level taxonomic distribution in hindgut samples at birth in most-efficient (M-eff) and least-efficient (L-eff) heifer calves. Figure S2. Non-metric multidimensional scaling (NMDS) plot of fecal microbiome profiles during the preweaning period in least-efficient (L-eff, n = 13) or most-efficient (M-eff, n = 13) heifer calves at (A) day 14, (B) day 28, and (C) day 42 of age. Figure S3. Phyla level taxonomic distribution in hindgut samples during the preweaning period at day 14, 28 and 42 of age in most-efficient (M-eff) and least-efficient (L-eff) heifer calves. Figure S4. Upregulated hindgut metabolites in M-eff heifer calves at birth strongly influencing metabolome discrimination between most-efficient (M-eff, n = 13) and least-efficient (L-eff, n = 13) heifer calves assessed by partial least square discriminant analysis (PLS-DA)l. Figure S5. Downregulated hindgut metabolites in M-eff heifer calves at birth strongly influencing metabolome discrimination between most-efficient (M-eff, n = 13) and least-efficient (L-eff, n = 13) heifer calves assessed by partial least square discriminant analysis (PLS-DA). Figure S6. Scores plot of partial least square discriminant analysis (PLS-DA) for hindgut metabolome profiles during the preweaning period in least-efficient (L-eff, n = 13) or most-efficient (M-eff, n = 13) heifer calves at (A) day 14, (B) day 28, and (C) day 42 of age. Figure S7. Upregulated hindgut metabolites in M-eff heifer calves during the preweaning period strongly influencing metabolome discrimination between most-efficient (M-eff, n = 13) and least-efficient (L-eff, n = 13) heifer calves assessed by partial least square discriminant analysis (PLS-DA). Figure S8. Downregulated hindgut metabolites in M-eff heifer calves during the preweaning period strongly influencing metabolome discrimination between most-efficient (M-eff, n = 13) and least-efficient (L-eff, n = 13) heifer calves assessed by partial least square discrimi [file 40104_2019_406_MOESM1_ESM.zip › Additional file 1 Fig. S8.jpg]
