# Supplementary material for: Residual feed intake divergence during the preweaning period is associated with unique hindgut microbiome and metabolome profiles in neonatal Holstein heifer calves
Source: J Anim Sci Biotechnol. 2020 Jan 20;11:13. doi: 10.1186/s40104-019-0406-x (PMC6972010; doi:10.1186/s40104-019-0406-x)
Supplement: Supplementary file 2 — Additional file 2: Table S1. Nutrient composition and amino acid profiles (mean ± standard deviation) of milk replacer (Advance Excelerate, Milk Specialties, Carpentersville, IL, USA) and starter grain (Ampli-Calf Starter 20; Purina Animal Nutrition, Shoreview, MN, USA) fed during the preweaning period to the most-efficient (M-eff, n = 13) or least-efficient (L-eff, n = 13) heifer calves. Table S2. Chemical taxonomy of top metabolites strongly influencing discrimination assessed by partial least squares discriminate analysis (PLS-DA) that were upregulated and downregulated in the hindgut of most-efficient (M-eff, n = 13) heifer calves compared with least-efficient (L-eff, n = 13) heifer calves at birth, following the conditions of VIP > 1.0 and |p-(corr)|. [file 40104_2019_406_MOESM2_ESM.docx]

**Table S1.** Nutrient composition and amino acid profiles (mean ± standard deviation) of milk replacer (Advance Excelerate, Milk Specialties, Carpentersville, IL, USA) and starter grain (Ampli-Calf Starter 20; Purina Animal Nutrition, Shoreview, MN, USA) fed during preweaning period to the most-efficient (M-eff, n = 13) or least-efficient (L-eff, n = 13) heifer calves.

| Item | Milk replacer | Starter grain mix |
| --- | --- | --- |
| Dry matter, g/kg | 946±11 | 908±27 |
| Crude protein, g/kg | 272±44 | 209±23 |
|  |  |  |
|  |  |  |
| Essential amino acids, % of DM |  |  |
| Arginine | 0.84±0.07 | 1.39±0.03 |
| Histidine | 0.59±0.02 | 0.55±0.01 |
| Isoleucine | 1.60±0.06 | 0.82±0.01 |
| Leucine | 2.90±0.07 | 1.52±0.03 |
| Lysine | 2.30±0.12 | 1.07±0.02 |
| Methionine | 0.53±0.02 | 0.29±0.01 |
| Phenylalanine | 1.01±0.02 | 0.98±0.01 |
| Threonine | 1.83±0.04 | 0.77±0.02 |
| Valine | 1.59±0.03 | 0.97±0.01 |
| Non-essential amino acids, % of DM |  |  |
| Aspartate | 2.89±0.07 | 2.00±0.05 |
| Alanine | 1.36±0.03 | 0.97±0.02 |
| Cysteine | 0.64±0.02 | 0.34±0.01 |
| Glutamate | 4.68±0.15 | 1.07±0.02 |
| Glycine | 0.60±0.03 | 0.98±0.01 |
| Proline | 1.64±0.04 | 1.14±0.02 |
| Serine | 1.47±0.05 | 0.99±0.03 |

**Table S2.** Chemical taxonomy of top metabolites strongly influencing discrimination by the partial least squares discriminate analysis (PLS-DA) that were upregulated and downregulated in hindgut in most-efficient (M-eff, n = 13) heifer calves compared with least-efficient (L-eff, n = 13) heifer calves at birth, following the conditions of VIP >1.0 and |p-(corr)| ≥ 0.5.

|  | Name | Chemical taxonomy | |
| --- | --- | --- | --- |
|  |  | Super class | Sub class |
| Upregulated in M-eff | 2,4-Dichlorophenol | Benzenoids | Halobenzenes |
|  | Prostaglandin F2a | Lipids and lipid-like molecules | Eicosanoids |
|  | Cervonoyl ethanolamide | Lipids and lipid-like molecules | Fatty acid esters |
|  | 9,10-DHOME | Lipids and lipid-like molecules | Fatty acids and conjugates |
|  | Muconic acid | Lipids and lipid-like molecules | Fatty acids and conjugates |
|  | 2-Lysolecithin | Lipids and lipid-like molecules | Glycerophosphocholines |
|  | Cholesterol ester | Lipids and lipid-like molecules | Steroid esters |
|  | 15(S)-HPETE | Lipids and lipid-like molecules | Eicosanoids |
|  | 2-Arachidonylglycerol | Lipids and lipid-like molecules | Endocannabinoids |
|  | AICAR | Nucleosides, nucleotides, and analogues | 1-ribosyl-imidazolecarboxamides |
|  | Nicotinamide ribotide | Nucleosides, nucleotides, and analogues | Nicotinamide nucleotides |
|  | Guanosine triphosphate | Nucleosides, nucleotides, and analogues | Purine ribonucleotides |
|  | 5-Aminolevulinic acid | Organic acids and derivatives | Amino acids, peptides, and analogues |
|  | Glucuronide | Organic oxygen compounds | Carbohydrates and carbohydrate conjugates |
|  | Biotin | Organoheterocyclic compounds | Biotin and derivatives |
| Downregulated in M-eff | Chenodeoxyglycocholate | Lipids and lipid-like molecules | Bile acids, alcohols and derivatives |
|  | Ceramide (d18:1/16:0) | Lipids and lipid-like molecules | Ceramides |
|  | Ceramide (d18:1/18:0) | Lipids and lipid-like molecules | Ceramides |
|  | Diglyceride | Lipids and lipid-like molecules | Diradylglycerols |
|  | Arachidonic acid | Lipids and lipid-like molecules | Fatty acids and conjugates |
|  | Alpha-dimorphecolic acid | Lipids and lipid-like molecules | Lineolic acids and derivatives |
|  | Pregnenolone | Lipids and lipid-like molecules | Pregnane steroids |
|  | Calcitriol | Lipids and lipid-like molecules | Vitamin D and derivatives |
|  | Cyclic GMP | Nucleosides, nucleotides, and analogues | Cyclic purine nucleotides |
|  | UDP-N-acetylglucosamine | Nucleosides, nucleotides, and analogues | Pyrimidine nucleotide sugars |
|  | Sphingosine | Organic nitrogen compounds | Amines |
|  | Chondroitin sulfate | Organic oxygen compounds | Carbohydrates and carbohydrate conjugates |
|  | Galactaric acid | Organic oxygen compounds | Carbohydrates and carbohydrate conjugates |
|  | L-Urobilin | Organoheterocyclic compounds | Bilirubins |
|  | Folinic acid | Organoheterocyclic compounds | Pterins and derivatives |

**Table S3.** Chemical taxonomy of top metabolites strongly influencing discrimination by the partial least squares discriminate analysis (PLS-DA) that were upregulated and downregulated in hindgut during preweaning period in most-efficient (M-eff, n = 13) or least-efficient (L-eff, n = 13) heifer calves, following the conditions of VIP >1.0 and |p-(corr)| ≥ 0.5.

|  | Name | Chemical taxonomy | |
| --- | --- | --- | --- |
|  |  | Super class | Sub class |
| Upregulated in M-eff | 8-Isoprostane | Lipids and lipid-like molecules | Eicosanoids |
|  | 15-KETE | Lipids and lipid-like molecules | Fatty acids and conjugates |
|  | Guanosine | Nucleosides, nucleotides, and analogues | Purine nucleosides |
|  | Deoxyuridine | Nucleosides, nucleotides, and analogues | Pyrimidine 2'-deoxyribonucleosides |
|  | S-Sulfo-L-cysteine | Organic acids and derivatives | Amino acids, peptides, and analogues |
|  | Fumaric acid | Organic acids and derivatives | Dicarboxylic acids and derivatives |
|  | Cytidine triphosphate | Organic oxygen compounds | Carbohydrates and carbohydrate conjugates |
|  | Biotin | Organoheterocyclic compounds | Biotin and derivatives |
|  | Ascorbic acid | Organoheterocyclic compounds | Furanones |
|  | Melatonin | Organoheterocyclic compounds | Indoles |
|  | L-Tryptophan | Organoheterocyclic compounds | Indolyl carboxylic acids and derivatives |
|  | (R)-lipoic acid | Organoheterocyclic compounds | Lipoic acids and derivatives |
|  | 5,10-Methylene-THF | Organoheterocyclic compounds | Pterins and derivatives |
|  | Pyridoxal phosphate | Organoheterocyclic compounds | Pyridine carboxaldehydes |
|  | Oxitriptan | Organoheterocyclic compounds | Tryptamines and derivatives |
| Downregulated in M-eff | Hippuric acid | Benzenoids | Benzoic acids and derivatives |
|  | Androstanedione | Lipids and lipid-like molecules | Androstane steroids |
|  | Glycocholic acid | Lipids and lipid-like molecules | Bile acids, alcohols and derivatives |
|  | Leukotriene E4 | Lipids and lipid-like molecules | Eicosanoids |
|  | 11Z-Eicosenoic acid | Lipids and lipid-like molecules | Fatty acids and conjugates |
|  | Oleic acid | Lipids and lipid-like molecules | Fatty acids and conjugates |
|  | LysoPC(P-18:1(9Z)) | Lipids and lipid-like molecules | Glycerophosphocholines |
|  | LysoPC(22:2(13Z,16Z)) | Lipids and lipid-like molecules | Glycerophosphocholines |
|  | LysoPC(18:3(6Z,9Z,12Z)) | Lipids and lipid-like molecules | Glycerophosphocholines |
|  | CE(18:0) | Lipids and lipid-like molecules | Steroid esters |
|  | CE(22:2(13Z,16Z)) | Lipids and lipid-like molecules | Steroid esters |
|  | LysoPC(22:1(13Z)) | Not classified | Not classified |
|  | Orotidine | Nucleosides, nucleotides, and analogues | Pyrimidine nucleosides |
|  | Maltotriose | Organic oxygen compounds | Carbohydrates and carbohydrate conjugates |
|  | D-Urobilin | Organoheterocyclic compounds | Bilirubins |
